# Assessing the comparability of toxic emissions reduction from heated tobacco aerosols relative to cigarette smoke: a scientific approach to bridging datasets

**DOI:** 10.1007/s11739-025-04160-6

**Published:** 2025-10-29

**Authors:** Jacqueline Miller-Holt, Grant O’Connell, Renaud Bach, Maurane Charriere, Yuki Kanemaru, Ziyi Su, Sylvain Larroque, Karin Jacobson

**Affiliations:** 1 JTI SA, 8 Rue Kazem Radjavi, 1202 Geneva, Switzerland; 2 Tobacco Science Research Center, Japan Tobacco Inc., 6-2, Umegaoka, Aoba-ku, Yokohama, Kanagawa 227-8512 Japan

**Keywords:** Emissions, Statistics, Heated tobacco, Bridging, Biomarker of exposure, Harm reduction

## Abstract

Heated tobacco products (HTPs) offer a reduced risk potential by avoiding combustion, thereby lowering toxic emissions. This study evaluated 34 heated tobacco stick (HTS) variants, each with different tobacco blends, using two heating devices to assess the comparability of 51 toxic emission reductions compared to combustible cigarette smoke. Aerosols and 1R6F reference cigarette smoke were generated under standardized conditions, and droplet or particle size distributions were measured. Toxic emission levels were analyzed using a proposed statistical approach to determine comparability. In vitro toxicological evaluation was performed using mutagenicity, genotoxicity, and cytotoxicity assays. Additionally, published data on Biomarkers of Exposure (BoE) were reviewed to assess whether reduced emissions corresponded to reduced human exposure at the category level. Overall, HTS emissions were reduced by 93.57% compared to cigarette smoke, with consistent reductions across blends and devices. HTS aerosols consistently showed significantly reduced in vitro biological activity compared to cigarette smoke, with reductions observed across all HTS types, devices, and sample phases, even at higher concentrations than those used for cigarette samples. Toxic emissions data from other HTP technologies in published literature were also reviewed, showing comparable reduced levels leading to consistent reductions in BoE. These findings suggest that the heat-not-burn principle provides substantial exposure reduction independent of product-specific attributes. The study supports the bridging of aerosol chemistry and toxicological datasets, and leveraging published BoE results across the HTP category, to streamline product assessments.

**Supplementary Information:**

The online version contains supplementary material available at 10.1007/s11739-025-04160-6.

## Introduction

Combustion of tobacco, which occurs at temperatures exceeding 400 °C and can reach up to 950 °C, produces a chemically complex mixture (smoke) containing thousands of constituents, including numerous toxicants known to be carcinogenic or harmful to respiratory and cardiovascular health [1, 2]. Epidemiological data confirm that the health risks of smoking are driven by sustained and long-term exposure to these primarily combustion-derived toxicants [3].

Technological innovation has led to the development of non-combustible nicotine products such as heated tobacco products (HTPs), which aim to reduce exposure to toxic emissions by eliminating combustion [4]. HTPs heat tobacco through controlled thermal processes that produce a respirable aerosol via evaporation and distillation rather than combustion [5, 6]. As a result, they do not generate carbonaceous solid particles and deliver nicotine-containing aerosols that are chemically less complex and substantially lower in toxicant content than combustible cigarette smoke [7–9].

HTPs also maintain many behavioral and sensory aspects of the cigarette smoking experience such as inhalation, hand-to-mouth action, thereby supporting their adoption by adults who smoke as a potentially less harmful alternative. Clinical studies show that switching to HTPs consistently results in marked and sustained reductions in biomarkers of exposure (BoE) to select toxic emissions, including known carcinogens and cardiovascular and pulmonary toxicants [10–14]. These exposure reductions have been shown to be further associated with favorable changes in biomarkers with predictive validity for several smoking-related diseases across the HTP category [15].

The consistency of these published findings across HTP technologies indicates that the primary driver of reduced toxicant exposure is the avoidance of combustion and not specific features such as tobacco blend, additives, or heating design technology. While such factors may influence aerosol chemistry to an extent it is the non-combustion feature that yields consistent and substantial reductions in toxic emissions across the HTP category. As such, there is growing scientific and regulatory interest in whether toxic emissions from one HTP can be used to support assessments of others, provided comparability can be demonstrated, through an approach known as bridging [16, 17].

The present study was designed to assess whether the heat-not-burn mechanism yields comparable reductions in toxic emissions and toxicological responses across a wide range of heated tobacco stick (HTS) variants. Emissions of 51 select toxicants were quantified across 34 HTS variants and compared to levels in combustible cigarette smoke. For a subset of HTS, in vitro toxicological responses were assessed. A statistical methodology is proposed to assess the comparability of reduced toxic emissions thereby enabling an analysis of the extent to which reduced toxic emissions can be considered comparable across HTS variants and in turn which HTS variants can be considered for data bridging. In addition, the study contextualizes these findings by comparing reduced toxic emissions profiles from the tested HTS to published emissions data from HTPs using different heating technologies, further informing the applicability of bridging across the broader HTP category. Together, these efforts aim to provide a foundational scientific basis for bridging emissions, whilst also considering the consistency of reduced exposure marker trends in clinical assessments across the broader HTP category, reinforcing the role of product design anchored in the heat-not-burn principle as the consistent driver of comparable reduced toxicant exposure and likely future reduced risk.

## Materials and methods

### Combustible cigarette & HTS test articles

To study the properties of cigarette smoke and a consistent baseline for analysis, 1R6F reference cigarettes were used (University of Kentucky, Lexington, KY, USA).

Thirty-four predominantly commercially available HTS consumables (available in Europe and Asia Region) were evaluated, comprising 17 regular (tobacco), 8 non-menthol, and 9 menthol variants, representing a broad range of tobacco blends (manufacturer JTI). Two heating devices (referred to as Device A and Device B; marketed under the Ploom brand (manufacturer JTI) were used in this study. Both devices operate at temperatures below 400 °C, under the reported ignition temperature of tobacco and include a temperature control system which is designed to preserve the integrity of the heat-not-burn mechanism, ensuring consistent aerosol output through precisely regulated conditions [2]. The heating element is external to the HTS tobacco matrix, termed ‘outside heating’.

**Fig. 1:**
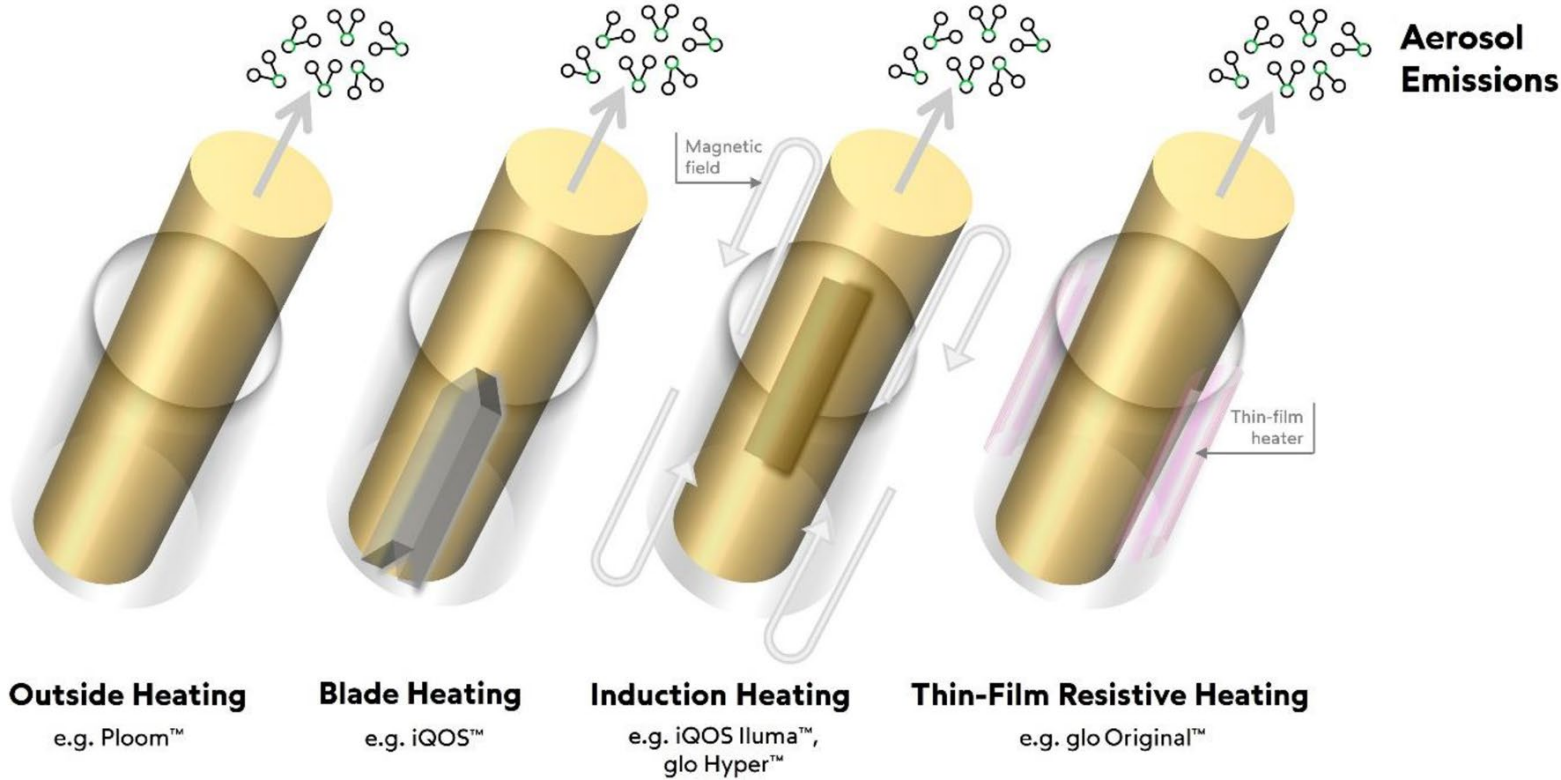
Schematic comparing different heating technologies: (external, blade, induction, and thin-film resistive) with corresponding heated tobacco stick (depicted as brown/gold cylinders) placement and direction of aerosol emissions release

To represent the diversity of thermal approaches in commercially available tobacco heating technologies, published aerosol chemistry data on devices employing blade [18, 19], induction [17, 20], and thin-film resistive [17] heating were used for comparisons (Fig. 1). For the physical measurements of aerosol droplet size in the present study, the induction heating-based device iQOS ILUMA, and its corresponding HTS consumable (TEREA) were used (manufacturer PMI). The device was operated according to the manufacturer instructions to ensure consistency and comparability with reported data.

### Smoke/aerosol generation & collection

HTS and 1R6F combustible cigarettes were conditioned as recommended in the International Organization for Standardization (ISO) 3402:2023 [21]. HTS aerosol was generated by machine puffing using a modified 20778:2018 puffing regime [22] now ISO 5501-1:2024 [23] (see Supplementary Materials (Table S1)).

### Chemical analysis of smoke & aerosol emissions

The yields of 51 toxic emissions were determined in aerosols produced by the HTS in combination with Device A^1^ or B and 1R6F cigarette smoke. These analytes include constituents suggested for the characterization of cigarette smoke by the scientific community and regulatory bodies (WHO [24], US FDA [25], Health Canada [26]). The assessed analytes also included the parent constituents corresponding to established BoEs associated with tobacco product use [27] and other constituents considered relevant to HTP [8, 9].

All analyte levels were determined from one batch of each HTS and tested in a minimum of three (max five) independent replicates. The mean and 95% confidence interval for each analyte is reported. A yield was considered below the detection limit (BDL) if the mean for three or five replicate analyses was below the limit of detection (LOD). A yield was classed as not quantified (NQ) if the mean for three or five replicate analyses was below the limit of quantification (LOQ) but above the LOD.

All chemical and toxicological analyses were conducted at ISO 17025 accredited laboratories, methods described in Supplementary Materials (Table S2).

### Physical measurements (particle/droplet release)

To characterize the droplet size distribution of aerosols generated by HTS with Device A or B, the commercial HTP comparator and particle size distribution of 1R6F cigarette smoke, a cascade impactor was employed (Mini-MOUDI 135-10, MSP Corporation), using ISO 20778:2018. The cascade impactor separated smoke/aerosol particles/droplets based on their aerodynamic diameters, allowing for a detailed characterization of the size distribution. Each sample was tested in six independent replicates.

### In vitro toxicological studies

The Ames, ivMN, and NRU assays were conducted in general accordance with the relevant Organization for Economic Co-operation and Development (OECD) guidelines and Health Canada official methods as previously described [7], with some modifications. Specifically, Ames and ivMN assays were conducted on both particulate and gas–vapor phases to broaden constituent coverage, despite Health Canada specifying particulate-phase testing only. The assays were performed with three independent replicates using three independent batches of the HTS aerosol or 1R6F cigarette smoke extract samples.

#### Sample preparation

HTS aerosols and 1R6F cigarette smoke were generated as described in Sect. “Smoke/aerosol generation & collection”. The particulate phase and gas–vapor phase of the test item aerosols/smoke were separately extracted and used in the in vitro assays. HTS aerosols were tested at maximal feasible concentrations (up to 10 × higher than 1R6F) to ensure maximum possibility of detectable biological activity.

#### Ames assay

Mutagenicity was assessed using Ames assay with *Salmonella typhimurium* strains TA98, TA100, TA102, TA1535, and TA1537. A test sample was classified as mutagenic if the result showed a reproducible concentration-related increase in the number of revertants and a statistically significant, as well as at least twofold for TA98, TA100, and TA102 and threefold for TA1535 and TA1537 increase in the number of revertants against the solvent control at one or more test concentrations [28].

#### In vitro micronucleus assay

Genotoxicity was assessed using ivMN assay with Chinese Hamster Ovary–Wolff–Bloom–Litton (CHO-WBL) cell line. A test sample was classified as genotoxic if it induced a reproducible concentration-related increase in the MN frequency, exceeded the historical solvent control range in the MN frequency, and showed a statistically significant increase in the MN frequency at one or more concentrations compared to the solvent control.

#### Neutral red uptake assay

Cytotoxicity was assessed using NRU assay performed with CHO-WBL cell line. Cell viability for each test sample treatment concentration was calculated as the relative absorbance to the solvent control.

### Estimation of comparability

#### Physical measurements

Mass median aerodynamic diameter (MMAD) and geometric standard deviation (GSD) of each sample were determined by fitting the data to a lognormal distribution, as described by the following equation:

$$\frac{{\mathrm{d}M}_{f}\left({d}_{p}\right)}{\mathrm{dln}{d}_{p}}=\frac{1}{\mathrm{ln}(\mathrm{GSD})\sqrt{2\uppi }}\mathrm{exp}\left[-\frac{{\left(\mathrm{ln}\left({d}_{p}\right)-\mathrm{ln}(\mathrm{MMAD})\right)}^{2}}{2{\left(\mathrm{ln}(\mathrm{GSD})\right)}^{2}}\right]$$

where ${M}_{f}\left({d}_{p}\right)$ is the normalized mass fraction and ${d}_{p}$ is the particle aerodynamic diameter.

The normalized mass fraction at each stage was divided by the logarithmic width of each stage, ∆lnD_50, which was computed from the midpoints of adjacent stage cut-off diameters. A non-linear least squares fitting was performed using the curve fit function from the SciPy. Optimize library in Python to obtain the MMAD and GSD.

#### Percentage reduction calculations aerosol versus reference cigarette smoke

Quantification results of the analytes are reported per tobacco stick/cigarette. Each individual BDL result was replaced with half the LOD, and each individual NQ result was replaced with (LOD + LOQ)/2.

The reduction of toxic emissions produced by HTS versus 1R6F cigarette was calculated using the following formula:

$${\text{Percent reduction}}=\frac{{\text{HTS mean}}-1{\mathrm{R}}6{\mathrm{F}} \ {\text{cigarette mean}}}{1{\mathrm{R6F}} \ \text{cigarette mean}}\times 100$$

where HTS mean value fell below the LOQ of the analytical method, a conservative approach was adopted and LOQ provided instead of the arithmetic mean. Where the HTS mean was below the LOD of the analytical method, the value of the LOD was used as mean. If an analyte was not quantified in 1R6F cigarette smoke (< LOQ), it was excluded from the yields comparison.

Having data generated at two accredited laboratories, we compared the relative differences of results to 1R6F combustible cigarette data produced within the same laboratory to mitigate any discrepancies.

#### Comparability of aerosol emissions

To evaluate the comparability in emissions reductions between the HTS aerosols, a statistical approach was employed using Horwitz–Thompson equations [29] to define comparability margins. This allows for the integration of cigarette-derived variability and measurement uncertainty into the comparison. Comparability was assessed using a two one-sided t test (TOST) procedure, applying a 5% significance level on each side (Fig. 4).

#### In vitro toxicological studies

For Ames and ivMN assay, the mutagenic and genotoxic potencies were estimated by the slope of the linear portion of the concentration–response curve using a generalized linear model with a Poisson distribution for tested samples that gave positive mutagenic or genotoxic responses. For NRU assay, cytotoxic potency was expressed using the half maximal inhibitory concentration (IC_50_) estimated from the concentration–response curve using the Hill function, a four-parameter logistic mathematical model, where the model was fit.

### BoE literature analysis and methodology for data aggregation

BoE data obtained from a previously published study with Device A (Nishihara et al. 2024) was reviewed to verify that the reduction in toxic emissions from HTS compared to cigarettes is reflected in human clinical data [10]. A PubMed® search (https://www.ncbi.nlm.nih.gov/pubmed/) was conducted in May 2025 to identify studies reporting BoE data following HTP use compared to cigarette smoking, using the search terms (“heated tobacco” AND “biomarkers of exposure”). In addition, a comprehensive published review on BoE data following HTP use by smokers (Akiyama and Sherwood 2021) was mined for relevant references [30]. Inclusion criteria were studies written in English, publications reporting data from randomized control trials and presentation of original data. Data reported in relevant studies were tabulated for analysis as percentage change in BoE levels compared to baseline.

For each biomarker, the mean percent change from baseline (with 95% confidence interval) was determined using the percentage changes as reported by or calculated from the included publications. The averaged percent change for Nishihara et al. (2024) was superimposed for visual comparability.

## Results

### Size of HTS aerosol droplets & smoke particles

Across the 34 HTS test articles (with aerosols generated by Device A and B), a commercially available induction heating HTP comparator and combustible cigarette smoke, the MMAD and GSD of the HTS aerosols and cigarette smoke were comparable, with all values falling within the respirable range (< 2.5 µm; Supplementary Materials (Table S3)).

### Reductions in levels of toxic emissions from HTS aerosols compared to cigarette smoke

**Fig. 2:**
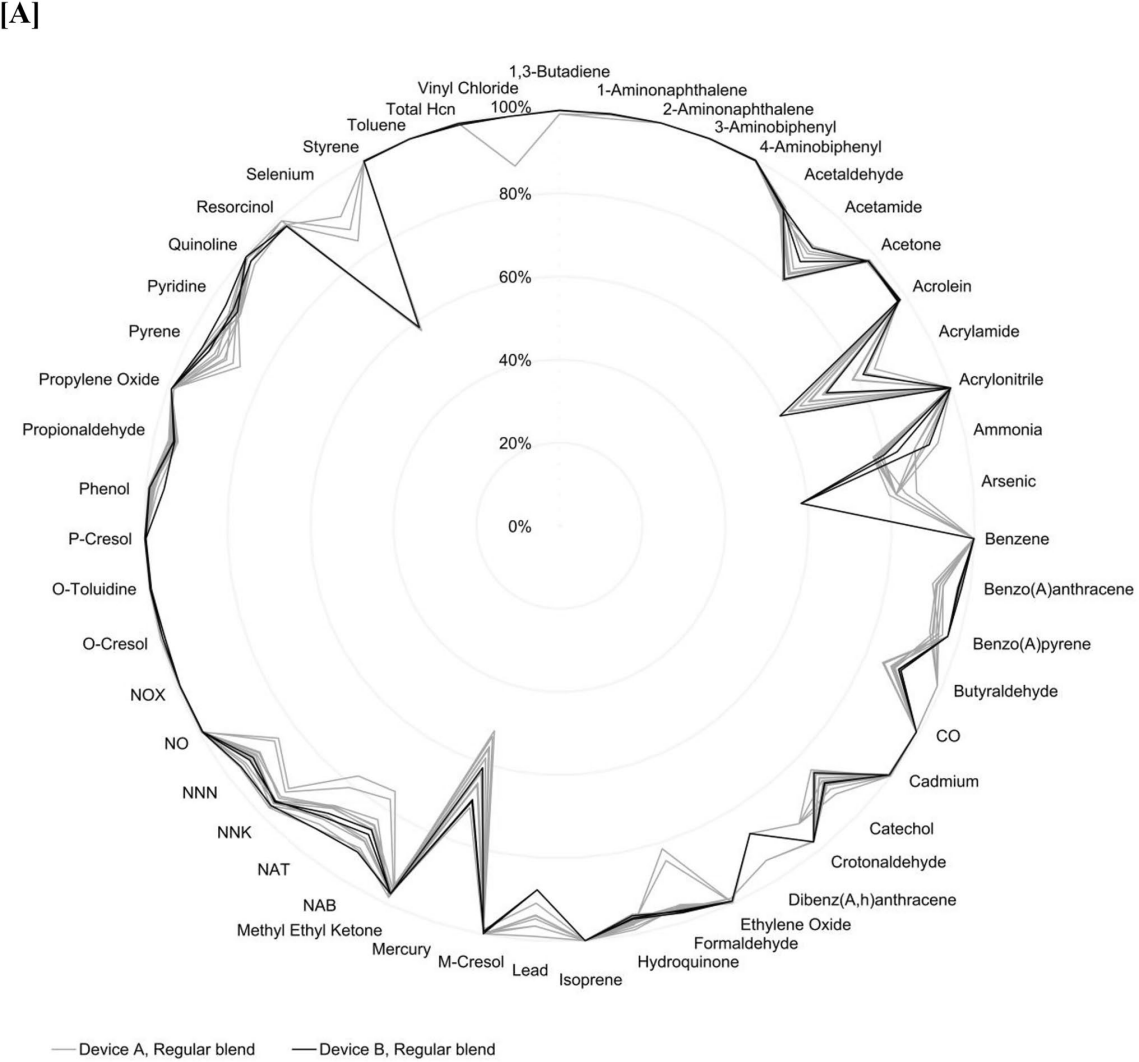

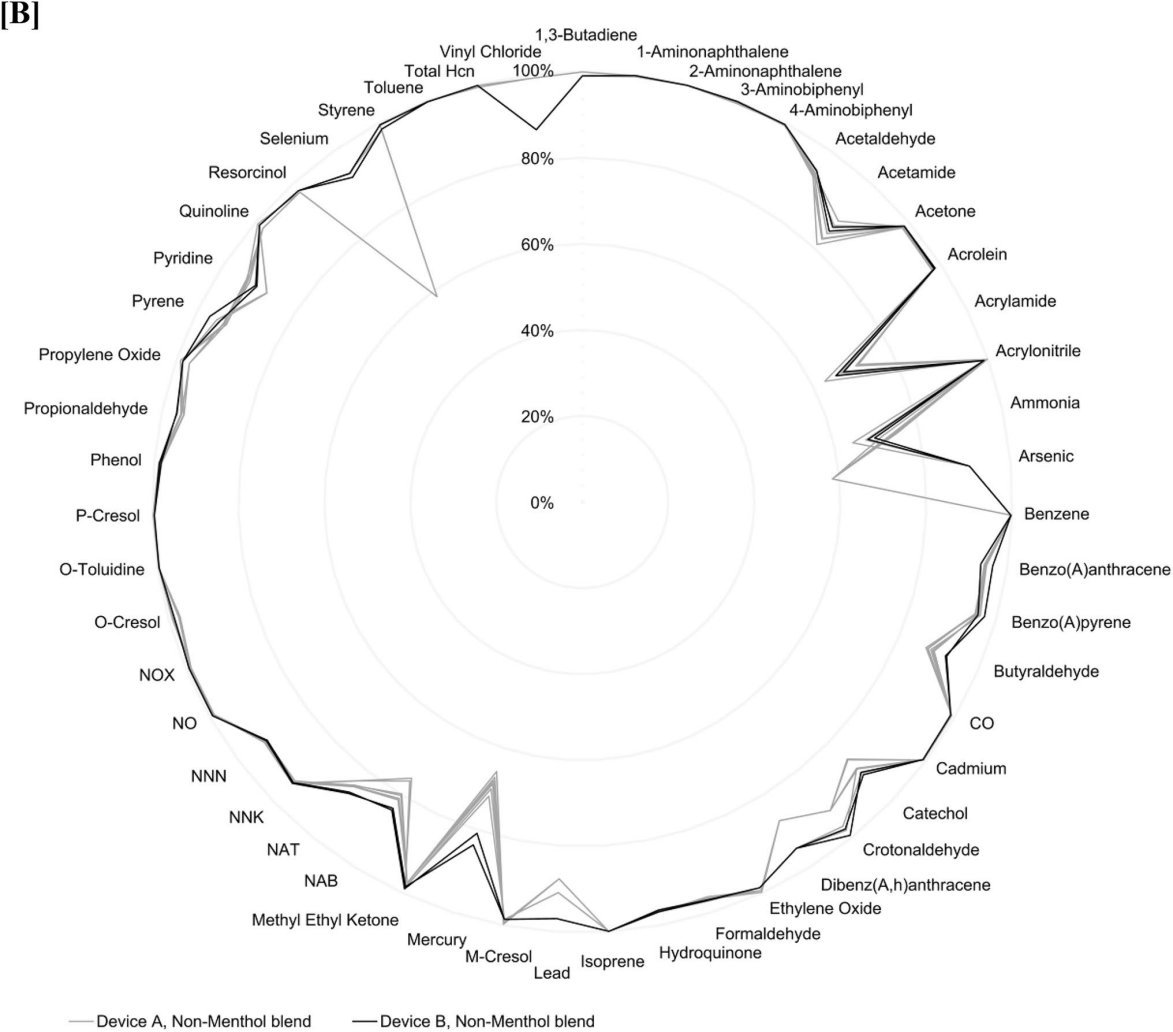

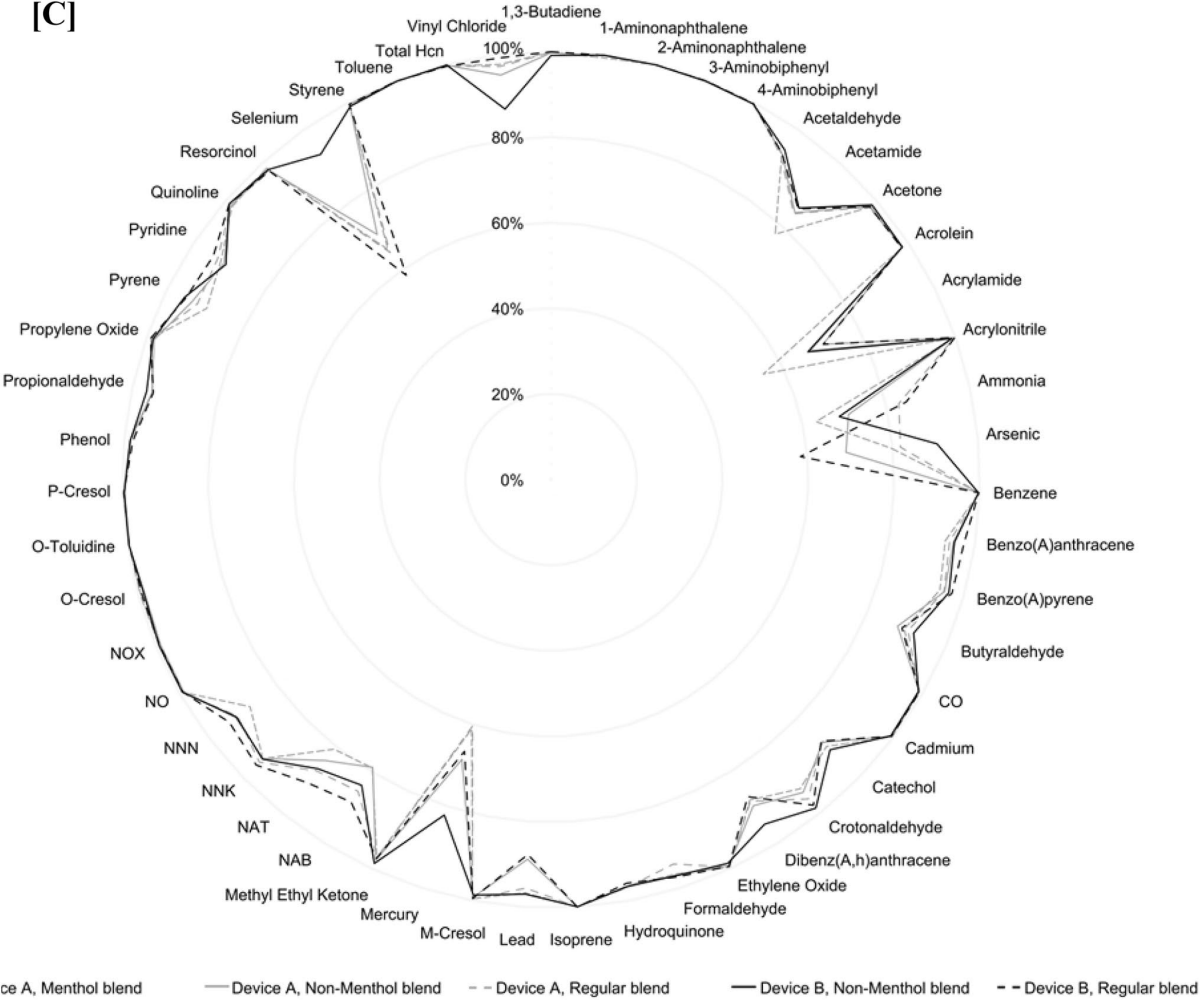
Percentage reduction in toxic emissions for **A** Regular blend HTS with Device A and Device B; **B** Non-menthol blend HTS with Device A and Device B; and **C** Menthol, non-menthol, and regular HTS blends with Device A and non-menthol and regular blend HTS with Device B. Reductions are calculated relative to combustible cigarette smoke for comparable analytes on a per-unit basis

Results showed substantial reductions in the levels of 51 toxic emissions across all 34 assessed regular, non-menthol, and menthol blend HTS, compared to cigarette smoke, whether using Device A or B (Fig. 2A and B and raw data provided in Supplementary Material). On a per-unit basis, levels of toxic emissions were consistently lower, with reductions ranging from at least 64% to over 99%, depending on the specific analyte measured. These reductions were consistently observed across the diverse set of HTS variants assessed, highlighting the comparability of emission reductions regardless of HTS product features such as blend, additives or device design [17–20].

As shown in the consolidated results in Fig. 2C, the use of Device A or Device B did not result in any meaningful difference in the mean percentage reduction for the toxic emissions analyzed.

**Fig. 3:**
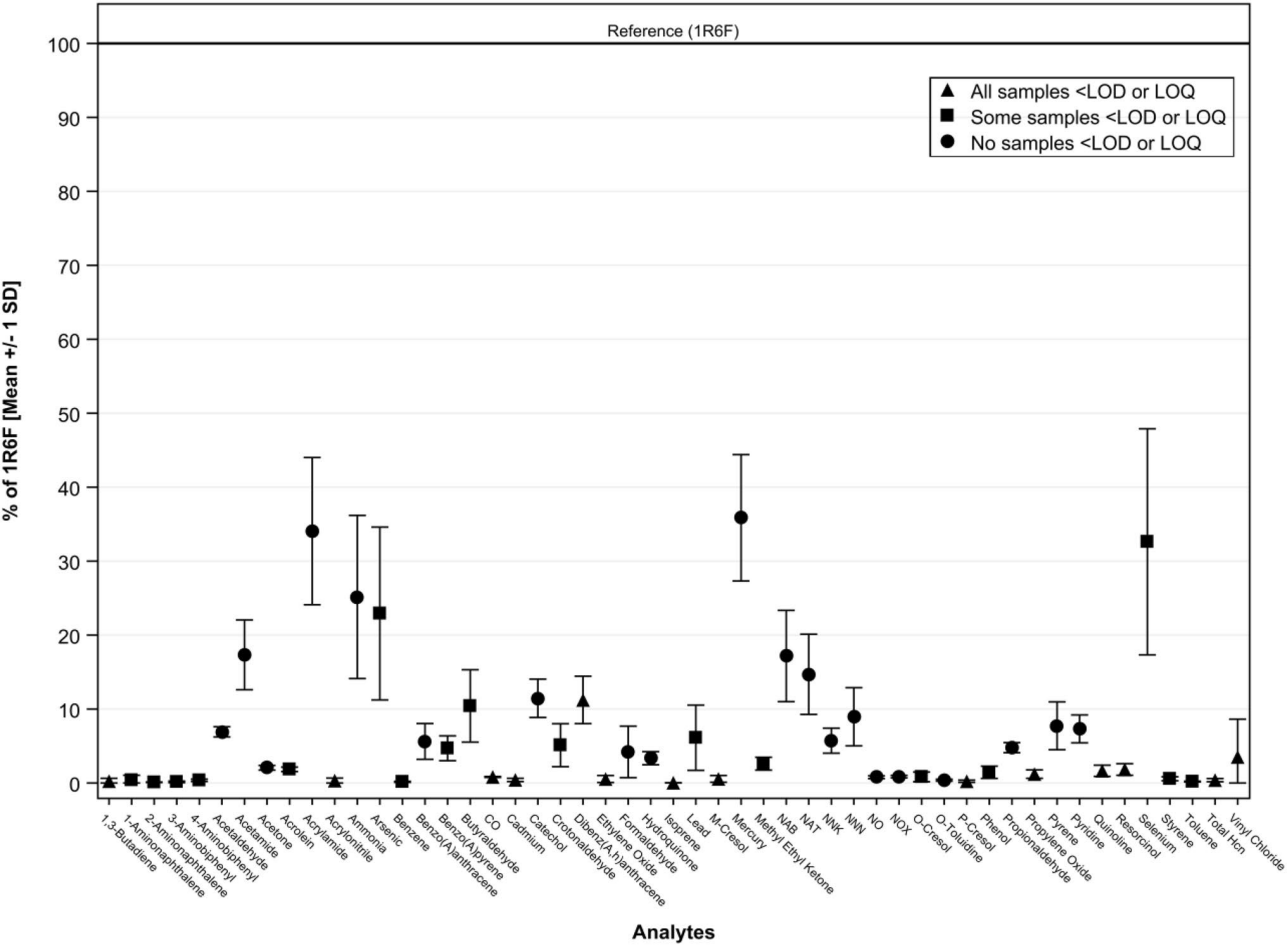
Comparison of mean reductions in toxic emissions for 34 HTS test articles using Device A and B compared to combustible cigarette smoke. Bar at 100% represents the 1R6F reference cigarette with values representing the average percentage of the 1R6F reference cigarette level across the measured analytes on a per-unit basis. Error bars indicate the standard deviation of pooled percentage

Figure 3 presents the mean percentage reduction in 51 toxic emissions across the 34 HTS relative to combustible cigarette smoke. Fourteen analytes (indicated with triangles) were consistently below the LOD or LOQ for both Device A and Device B. This group includes two analytes from the WHO Tobacco Product Regulation Study Group (TobReg) 9 list of priority toxicants (1,3-butadiene and carbon monoxide) [24], underscoring the potential for meaningful reductions in exposure. Seventeen analytes (indicated with squares) were below LOD or LOQ in some, but not all, of the HTS tested, while the remaining 20 analytes (indicated with circles) were consistently quantifiable across all HTS aerosols. For these quantifiable analytes, the standard deviation of mean values was low, reflecting reproducibility of reductions across products. Even in cases where variability was observed, the reductions relative to cigarette smoke remained substantial.

**Fig. 4:**
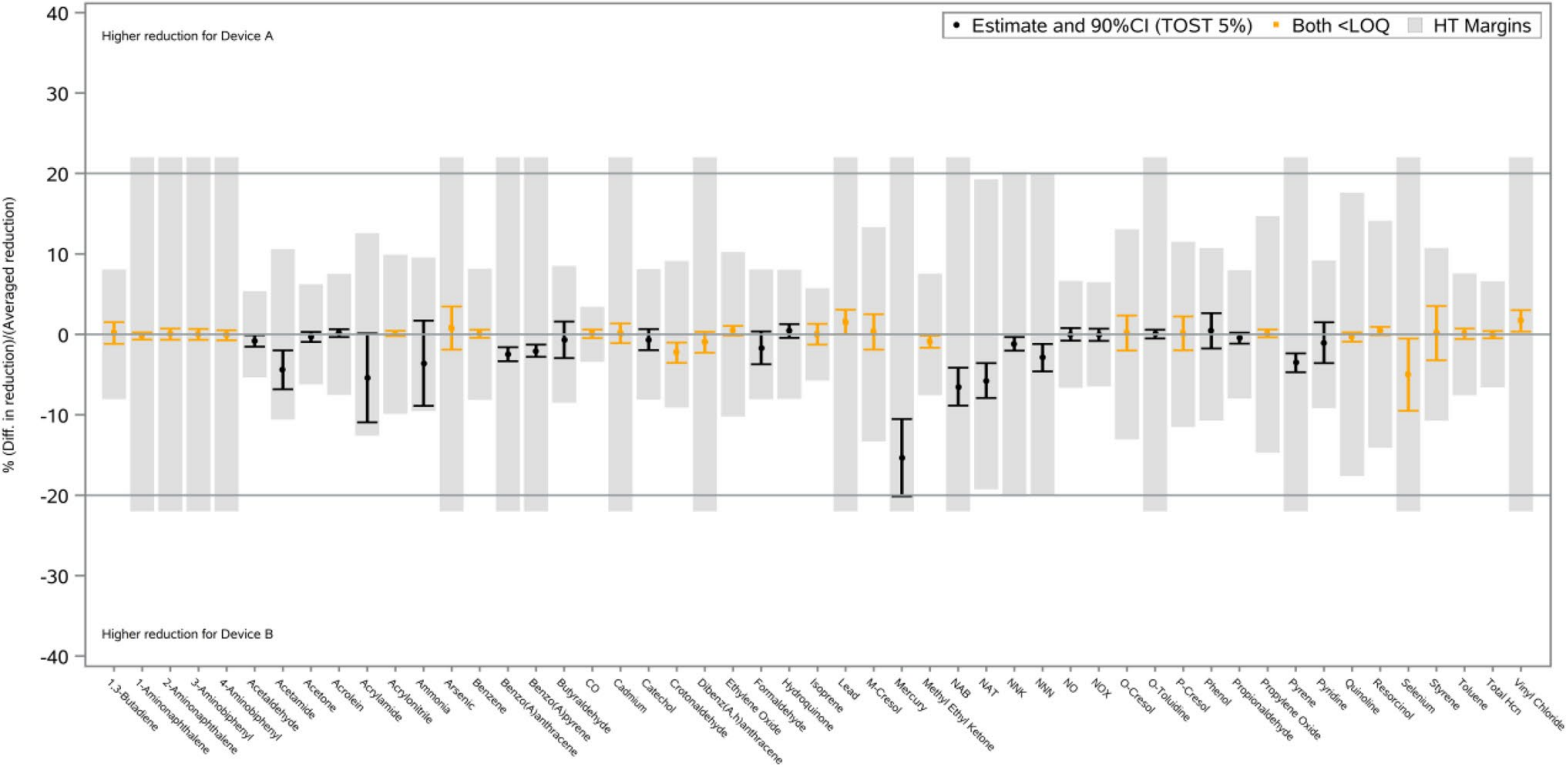
Statistical comparability testing (two-one-sided *t* tests [TOST], 5% level) of the difference in the 51 toxic emission reductions relative to 1R6F cigarette smoke for HTS assessed with Device A and Device B. Point estimates and 90% confidence intervals within Horwitz-Thompson (HT) margins

Figure 4 further confirms this pattern. Toxic emissions reduction values were tightly clustered around the median, demonstrating a high level of consistency across HTS aerosols. Statistical analysis showed that reductions were of comparable magnitude between Device A and Device B, with all values falling within an acceptable and scientifically relevant range.

### Reductions in levels of toxic emissions across heated tobacco technologies

A comparative analysis of HTS aerosols demonstrated that heated tobacco technologies, regardless of the heating mechanism employed, yield substantial reductions in toxic emissions relative to combustible cigarette smoke. Mean percentage reductions ranged from 62.47–99.98% for Device A, 69.29–99.9% for Device B, 51.2–99.9% for iQOS induction heating technology and 51.61–99.9% for iQOS blade heating technology.

Further assessment of heated tobacco products using alternative heating technologies, including published data on glo thin-film resistive heating and glo induction (in standard and boost modes) [17], and both blade and induction variants of iQOS, revealed comparable reductions in the WHO Tobacco Product Regulation Study Group (TobReg) 9 priority toxicants (Table 1). Across all evaluated heating technologies with published datasets, the overall reduction in these priority toxicants ranged from 96.47% for glo thin-film resistive heating, 95.67% for glo induction standard mode, 96.59% for glo induction boost mode, 95.10% for iQOS blade heating, 95.56% for iQOS induction heating, and 96.27% for the outside heating evaluated in this study. The reported maximum heating temperatures of the devices ranged from 240 to 350 °C.

**Table 1:** Comparison of percentage reductions in toxic emissions relative to reference cigarette smoke (1R6F or 3R4F), based on published data for induction heating, blade heating technologies or other heating technologies

Analyte (smoke/aerosol)	Mean % reduction HTP aerosol (Device A) vs. 1R6F cigarette smoke	Mean % reduction HTP aerosol (Device B) vs. 1R6F cigarette smoke	Mean % reduction HTP aerosol vs. 1R6F cigarette smoke [this study]	Reported data on iQOS™ Induction Heating Technology [20] HTS regular/HTS menthol	Reported data on iQOS™ blade heating technology [18, 19] HTS regular/HTS menthol	Reported data glo™ original thin-film resistive heating technology [17]	Reported data glo™ hyper induction heating technology (standard mode operation) [17]	Reported data glo™ hyper induction heating technology (boost mode operation) [17]
1-Aminonaphthalene	99.57	99.85	99.62	99.9/99.9	> 99.85/99.85	–	–	–
2-Aminonaphthalene	99.91	99.90	99.91	99.9/99.9	> 99.9/99.9	–	–	–
3-Aminobiphenyl	99.85	99.85	99.85	99.8/99.8	99.7^a^	–	–	–
4-Aminobiphenyl	99.62	99.68	99.63	99.6/99.6	99.72/99.64	–	–	–
Acetaldehyde	92.97	93.71	93.09	89.5/87.9	88.49/88.36	94.0	91.4	91.3
Acetamide	82.11	85.65	82.68	83.8/84.7	73.33/73.9	–	–	–
Acetone	97.89	98.18	97.94	95.4/95.5	95.48/95.34	–	–	–
Acrolein	98.16	98.14	98.16	94.4/94.6	94.8/94.71	98.5	98.1	98.4
Acrylamide	65.30	69.29	65.95	79.2/78.2	62.12/58.43	–	–	–
Ammonia	74.17	78.40	74.86	51.2/65.6	58.55/57.79	–	–	–
Arsenic	78.22	71.21	77.09	89.7/90.6	> 85.42/> 85.42	–	–	–
1,3-butadiene	99.79	99.57	99.76	99.9/99.9	99.75/99.71	> 99.9	> 99.9	> 99.9
Benzene	99.86	99.77	99.85	99.5/99.4	99.42/99.32	> 99.9	99.9	99.9
Benz[a]anthracene	93.97	96.54	94.38	95.7/94.7	91.41/93.72	–	–	–
Benzo(a)pyrene	95.01	96.90	95.31	96.2/95.5	93.13/95.63	> 97.4	> 97.8	> 98.4
Butyraldehyde	89.50	90.06	89.59	81.5/77.2	75.6^a^	–	–	–
Catechol	88.48	88.88	88.55	88.1/87.8	86.85/87.05	–	–	–
Crotonaldehyde	94.31	97.94	94.90	92.9/92.9	> 94.04/94.04	–	–	–
Carbon monoxide	99.20	99.10	99.18	99.1/99.2	> 99.78/> 99.78	> 99.5	> 99.3	99.1
Formaldehyde	95.52	97.21	95.80	90.9/91.1	89.89/89.06	95.2	95.2	95.8
Hydroquinone	96.75	96.19	96.66	91.4/92.2	91.9^a^	–	–	–
Lead	94.35	91.43	93.88	98.3/98.3	NA/NA	–	–	–
Mercury	62.47	72.83	64.14	72.7/68.0	51.61/56.88	–	–	–
Methyl Ethyl Ketone	97.29	97.92	97.39	97.0/96.0	96.15/96.14	–	–	–
NAB	82.09	86.70	82.84	88.9/89.1	93.6^a^	–	–	–
NAT	84.57	89.16	85.31	93.3/93.6	94.8^a^	–	–	–
NNK	94.14	94.97	94.27	95.5/96.2	96.12/97.02	96.8	95.9	97.8
NNN	90.64	93.20	91.05	95.0/96.7	94.51/96.57	87	83.5	88.7
NO	99.22	99.24	99.22	97.3/97.8	97.3^a^	–	–	–
NOx	99.14	99.21	99.15	97.3/97.8	97.5^a^	–	–	–
o-Cresol	99.23	98.93	99.18	99.0/98.8	98.91/98.88	–	–	–
o-Toluidine	99.63	99.59	99.62	99.1/99.1	98.97/98.97	–	–	–
Phenol	98.62	98.21	98.56	94.3/93.5	93.47/94.36	–	–	–
Propionaldehyde	95.17	95.59	95.23	91.3/90.2	90.24/90.08	–	–	–
Pyrene	91.69	95.29	92.27	93.9/93.0	–	–	–	–
Pyridine	92.61	93.09	92.69	83.9/84.0	79.3^a^	–	–	–
Selenium	66.45	72.29	67.39	80.8/80.8	NA/NA	–	–	–
Styrene	99.46	99.29	99.44	97.9/97.7	97.48/97.42	–	–	–
Toluene	99.82	99.76	99.81	98.9/98.8	99.02/98.85	–	–	–
Maximum device heating temperature (°C)	≤ 320			350	350	240	250	260

Percent reductions are not shown when the yields of the analyte in the aerosol from the 34 HTSs in combination with Device A or B are always below the limit of detection (LOD) or limit of quantification (LOQ)

^a^Levels for these analytes were referred to former publication on Tobacco heating System 2.2 (THS 2.2) [19]

### In vitro biological activity

Twenty-three HTS test items, representing different stick consumable varieties (regular, non-menthol, and menthol blends) in combination with two different devices (Device A and B), were assessed alongside the 1R6F cigarette. Both particulate phase (aerosol collected mass (ACM); HTS or total particulate matter (TPM); cigarette smoke) and the gas–vapor phase (GVP) samples from the HTS aerosols and cigarette smoke were evaluated.

#### Ames assay (mutagenicity assessment)

Detailed results for each individual HTS test item and 1R6F cigarette are provided in Supplementary Materials (Tables S4 and S5). The ACM and GVP samples from all HTS test items showed non-mutagenic responses under the test conditions. In contrast, the TPM sample from 1R6F cigarette induced clear and reproducible mutagenic responses in strains TA98 and TA1537 under + S9 conditions, while the GVP samples showed non-mutagenic responses under the test conditions. Due to the absence of mutagenic activity of the HTS test items, a direct comparative analysis with 1R6F cigarette was not pursued.

#### In vitro micronucleus assay (genotoxicity assessment)

Genotoxic potential was evaluated using the ivMN assay under the three treatment conditions: 3 h exposure without metabolic activation (Short − S9), a 3 h exposure with metabolic activation (short + S9), and 30 h exposure without metabolic activation (Long − S9). Detailed results, including genotoxicity assessments and genotoxic slope values for each HTS test item and 1R6F cigarette, are provided in Supplementary Materials (Table S6).

Most HTS samples were classified as genotoxic in at least one treatment schedule. However, several HTS samples, irrespective of HTS stick blend or device, did not induce statistically significant increases in MN frequency compared to solvent controls, and were, therefore, assessed as non-genotoxic under the test conditions. These inconsistencies are likely attributable to inherent biological variability associated with ivMN assay. Such variability may influence whether a weak response meets or fails to meet all genotoxicity evaluation criteria required for a positive call, especially in borderline cases.

In contrast, both TPM and GVP samples from 1R6F cigarettes demonstrated clear genotoxicity under all treatment conditions. Notably, the tested concentrations of the HTS test items were approximately six- to tenfold higher than those tested for the 1R6F cigarette. Overall, the genotoxic activity of HTS test items was reduced by 90% to 98% for ACM samples and from 86 to 96% for the GVP samples, relative to the 1R6F cigarette (see Supplementary Materials (Fig. S1)). Across the stick types, the percentage reduction in genotoxic activity relative to 1R6F cigarette was consistent and comparable (Fig. S1A, C, and E). Likewise, no significant differences were observed between the two device datasets (Fig. S1B, D, and F).

#### Neutral red uptake assay (cytotoxicity assessment)

Cytotoxicity was evaluated using NRU assay. IC_50_ values for each test sample from individual HTS test items and 1R6F cigarette are provided and compared in Supplementary Materials (Table S7 and Fig. S2).

The ACM samples from the individual HTS test items showed an 86% to 94% reduction in cytotoxicity compared to 1R6F cigarette TPM sample. Similarly, the GVP samples from the HTS test items demonstrated at least 93% reduction in cytotoxicity compared to the 1R6F cigarette GVP sample. These reductions in cytotoxic potential were consistent and comparable across all three HTS stick types (regular, non-menthol, and menthol; Fig. S2A) and between Device A and B (Fig. S2B).

### Trends in reduced biomarkers of exposure and reduced toxic emissions in aerosols

Biomarkers of exposure (BoE) provide a direct measure of user exposure to select substances that are quantifiable in HTP aerosols [27, 31]. Clinical assessments consistently demonstrate, independently of differences in HTP designs or characteristics, adults who smoke and who switch away from combustible cigarettes to HTP experience substantial and sustained reductions in BoEs relative to continuing to smoke [10, 14]. Moreover, the magnitude of these reductions is typically not dissimilar to those observed following smoking cessation, reinforcing the relevance of BoEs as indicators of reduced toxic emissions exposure.

**Fig. 5:**
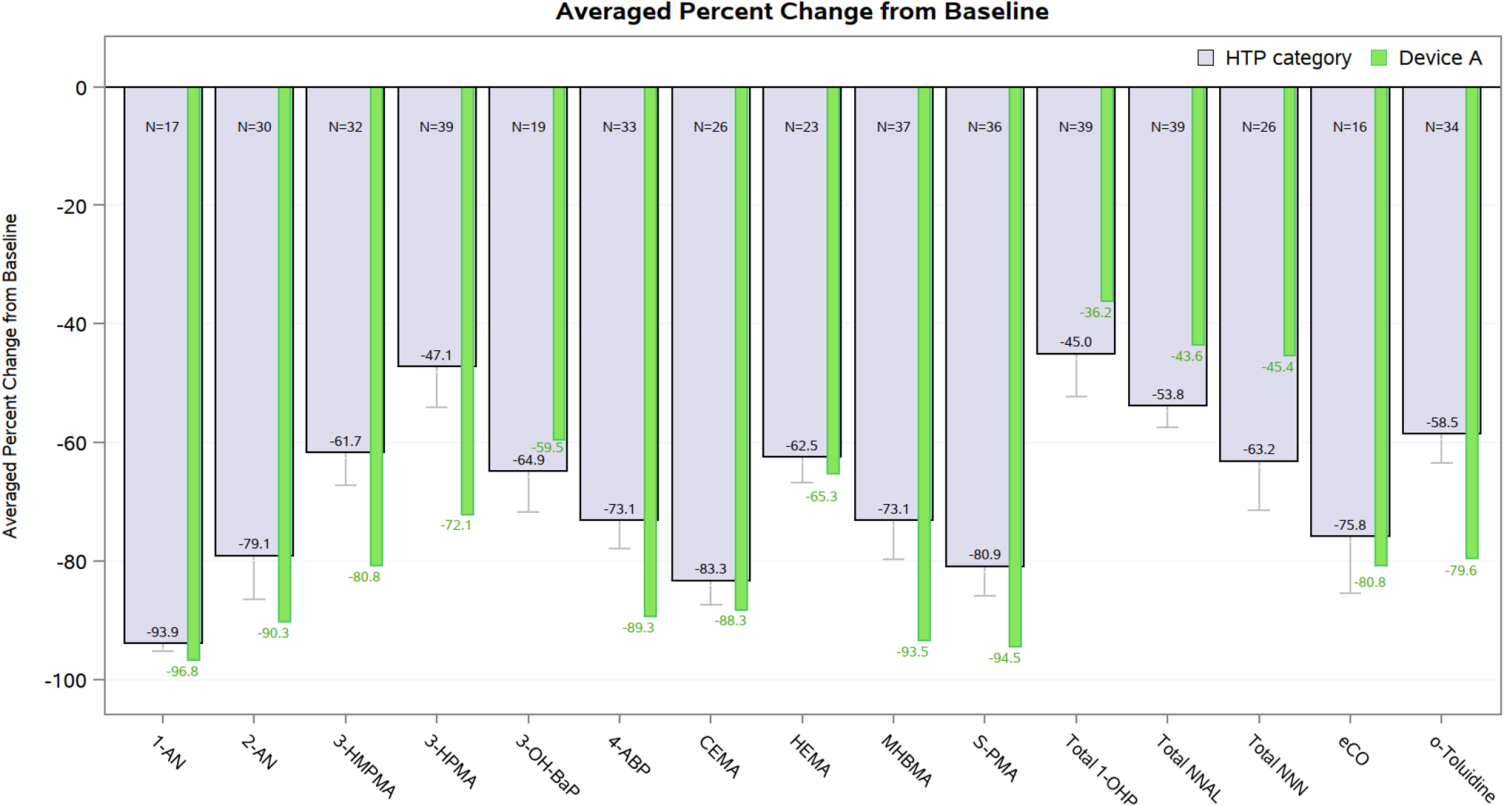
Consistency of BoE reductions: outside heating (Device A) in the context of category-level HTP data. Bars represent mean percent reductions from baseline with 95% confidence intervals of the mean percent reduction for each BoE among adult smokers randomized to use HTP. Category-level reductions (grey bars) are derived from a pooled analysis of 24 published studies. Number of individual study entries per biomarker are shown in text (*N* =). Results for outside heating technology (Device A/Nishihara et al., (2024)) overlaid in green bars in comparison with the HTP category trends. Acronyms for BoEs with corresponding parent compound in aerosol: 1-AN, 1-Aminonaphthalene (1-Aminonaphthalene); 2-AN, 2-Aminonaphthalene (2-Aminonaphthalene); 3-HMPMA, 3-hydroxy-3-methylpropylmercapturic acid (crotonaldehyde); 3-HPMA, 3-hydroxypropyl-mercapturic acid (acrolein); 3-OH-BaP, 3-hydroxy-benzo[a]pyrene (benzo[a]pyrene); 4-ABP, 4-Aminobiphenyl (4-Aminobiphenyl); CEMA, 2-cyanoethylmercapturic acid (acrylonitrile); HEMA, 2-hydroxyethyl-mercapturic acid (ethylene oxide); MHBMA, monohydroxybutenyl-mercapturic acid (1,3-butadiene); S-PMA, S-phenylmercapturic acid (benzene); Total 1-OHP, Total 1-hydroxypyrene (pyrene); Total NNAL, Total 4-(methylnitrosamino)-1-(3-pyridyl)-1-butanol, (4-(methylnitrosamino)-1-(3-pyridyl)-1-butanone [NNK]); Total NNN, Total N-nitrosonornicotine (N-nitrosonornicotine [NNN]); eCO, exhaled carbon monoxide (carbon monoxide); o-Tol, o-Toluidine (o-Toluidine)

Previous clinical data for Device A demonstrated reductions in 15 commonly assessed BoE after 5 days of switching from cigarettes [10]. To contextualize these findings, a pooled analysis of data from published clinical studies on the same 15 BoEs assessed among adult smokers randomized to use HTP across 24 studies, covering exposure durations from 5 days to 1 year and encompassing a range of heating technologies was conducted (see Supplementary Materials (Table S8)) for information on the included studies) [10–15, 27, 31–48]. Despite wide variation in study designs (e.g. duration, participants number, location) and reported measures (e.g. geometric mean least squares mean), pooled data analysis enabled the identification of trends, including average reduction and inter-study variability for each BOE. The pooled results, represented an average percent change from baseline, indicate overall consistent reductions in BoEs at the HTP category-level ranging from 45% to 93.9%, irrespective of device design, study duration, or study location (Fig. 5). As illustrated in Fig. 5, the trajectory of BoE reductions from baseline observed with outside heating technology (Device A), ranging from 36.2% to 96.8%, closely aligns with overall HTP category BoE trends.

## Discussion

This study evaluated the hypothesis that adherence to the core principle of heating rather than burning tobacco consistently yields substantially lower levels of toxic emissions that are comparable across HTPs, irrespective of differences in product design, tobacco blend, additive composition, or heating technology, thereby providing a foundational scientific basis for bridging datasets across the HTP category based on the comparability of reduced toxic emissions within a statistically acceptable range.

To assess this, 34 HTS variants were evaluated for levels of toxicant emissions and, for a subset of HTS, the resulting in vitro biological activity response. Across all products, emissions of 51 toxic emissions were substantially reduced (average of 93.6%) compared to cigarette smoke. A proposed statistical analysis approach confirmed that the reductions fell within an acceptable range of comparability. Despite technological diversity, these findings aligned with published data from other commercial HTPs (Table 1), reinforcing reproducibility and reliability of toxic emissions reductions at the category level [17–20]. For constituents with less pronounced average reductions, i.e. for selenium, arsenic, acrylamide, ammonia and mercury, levels remained within documented levels from comparable products [18, 20]. Notably, the majority of arsenic and selenium results were below LOD or LOQ, with detectable levels in only 3–4 HTS compared to 1R6F (arsenic: 3/34; selenium 4/34). These five analytes were present at negligible levels in aerosols relative to cigarette smoke and consistent with the published data (Supplementary Materials (Table S9)). Importantly, in vitro toxicology confirmed significantly lower overall toxicity for HTS, despite test concentrations being up to 7.3 × higher than those of 1R6F smoke on a nicotine-equivalent basis.

The biological relevance of these toxic emissions reductions was confirmed through three standard in vitro assays. For the subset of tested products, HTS was non-mutagenic in the Ames assay under the test conditions, contrasting with the mutagenic response observed for cigarette smoke particulate matter. Genotoxicity, as measured by the in vitro micronucleus (ivMN) assay, was reduced by 86–98%, and cytotoxicity, assessed via the neutral red uptake (NRU) assay, was reduced by 86–94%. These consistent reductions in biological activity indicate that reduced toxic emission yields translate into a meaningful reduction in toxicological risk potential.

These findings confirm that comparable reductions in toxic emissions and toxicological activity are functionally relevant and attributable to the shared design principle of avoiding combustion. This shared mechanism serves as the foundation for considering data bridging across HTPs [16].

In parallel, aerosol droplet size distribution was also measured to exclude differences in delivery efficiency as a confounding factor. HTS produced aerosols with mass median aerodynamic diameters (MMADs) < 2.5 µm, confirming respirability. These values were comparable to a commercial HTP comparator and cigarette smoke, indicating observed reductions in toxicological activity can be attributed to differences in aerosol chemistry, not delivery characteristics or deposition efficiency.

Biomarkers of exposure (BoE) provide a direct and quantitative measure of exposure to harmful substances and are closely linked to the levels of toxic emissions present in product aerosol. Given that all HTPs operate on the same fundamental principle of heating rather than burning tobacco, they consistently generate substantially lower emissions than combustible cigarettes. Therefore, it follows that BoE trends are expected to align at the product-category level, independent of individual device design, tobacco blend, or additives.

Consistent with the published literature, complete switching away from combustible cigarettes to HTPs leads to substantial reductions in BoE, with reductions for outside heating technology (Device A) ranging from 36.2% to 96.8% after 5 days of exclusive use [10–14]. When considered alongside the pooled data from 24 published studies across different heating technology devices, exposure durations and geographies, a clear pattern emerges: BoE reductions cluster at the HTP category level with reductions achieved with outside heating technology reflecting the broader category effect rather than being product-specific.

Importantly, this pattern is reinforced by aerosol chemistry. Comparability of reduced toxic emissions (Fig. 4) leads to BoE readouts that sit within category-level trends (Fig. 5), indicating that similarities in aerosol toxic emissions translate into consistent user exposure reductions across different heating technologies. The mechanistic basis for this consistency lies in the absence of combustion across all HTPs, which eliminate or markedly reduce the formation of many harmful substances that are characteristic of cigarette smoke. Therefore, while heating technologies may vary in design, the fundamental absence of combustion provides a unifying explanation for comparable reductions in toxic emissions in aerosols and corresponding reductions in user exposure. This study supports linking product-specific toxic emissions profiles with the broader evidence base on BoE across HTPs. Thus, consistency in thermal operations provides a robust scientific basis for evaluating reduced toxic emissions comparability and for bridging these datasets across HTPs, regardless of individual product design provided the core thermal principle is preserved, underpinned by clinical outcomes observed at the HTP category level.

Although our analysis demonstrated consistent reductions in BoE trends across HTP technologies, translation of these exposure changes into direct human health outcomes was not assessed. The inference that reduced toxic emissions leads to reduced exposure, and likely reduced risk, is based on established evidence linking lower toxicant exposure to health outcomes in smokers who reduce combustion exposure. While scientifically reasonable and aligned with toxicological and epidemiological principles, confirmation requires long-term data. In time, such evidence will emerge at the category level, reflecting the shared absence of combustion that defines the category and drives the consistent reductions in exposure observed to date. Product-specific evaluations of the comparability of reduced toxic emissions, such as in this study, help build the mechanistic and empirical foundation for future category-wide epidemiology. These studies also contribute to a broader framework for assessing the harm reduction potential, though population-level impact will ultimately depend on broad HTP adoption and sustained switching from cigarettes.

Bridging of emissions and toxicology datasets is a well-established scientific and regulatory practice. Frameworks such as the European Chemicals Agency’s (ECHA) read-across strategy and the UK Medicines and Healthcare products Regulatory Agency (MHRA)’s guidance on representative e-cigarette submissions endorse the use of representative data where comparability can be scientifically justified [49, 50]. Across all HTS tested in the present study, toxic emissions and in vitro responses were consistently and substantially lower than those of combustible cigarettes, with a proposed statistical analysis approach confirming the comparability of toxic emissions reductions. Comparability of reduced toxic emissions also leads to BoE readouts that sit within category-level trends.

Overall, this work provides a strong scientific basis for bridging assessments across HTP innovations by demonstrating consistent and comparable reductions in toxic emissions. These findings also support a category-level approach to evaluating harm reduction and facilitate integration of product-specific data with future epidemiological evidence.

## Supplementary Information

Below is the link to the electronic supplementary material.

Supplementary file1 (DOCX 2737 KB)

Supplementary file2 (XLSX 44 KB)

## Data Availability

The datasets produced and analysed in this study are provided within the supplementary information. Additional information can be obtained from the corresponding author upon request.

## Abbreviations

HTP: Heated tobacco products
HTS: Heated tobacco stick
ACM: Aerosol collected matter
GVP: Gas vapour phase
BDL: Below the detection limit
LOD: Limit of detection
LOQ: Limit of quantification
NQ: Not quantified
ivMN: In vitro micronucleus
NRU: Neutral red uptake
TPM: Total particulate matter
MMAD: Mass median aerodynamic diameter
GSD: Geometric standard deviation
TOST: Two one-sided t-test
ECHA: European Chemical Agency
MHRA: UK Medicines and Healthcare products Regulatory Agency
HT Margins: Horwitz–Thompson margins
OECD: Organization for Economic Co-operation and Development
CHO-WBL: Chinese Hamster Ovary–Wolff–Bloom–Litton
BoE: Biomarkers of exposure

## Footnotes

1 Device A refers to minor incremental versions of the same device, with the maximum heating temperature of each not exceeding 320 °C.

## References

[1] Baker RR (1981) Product formation mechanisms inside a burning cigarette. Prog Energy Combust Sci 7(2):135–153. 10.1016/0360-1285(81)90008-3

[2] Bechikhi M, Masson E, Herbinet O, Dufour A (2024) Mapping of tobacco conversion characteristics in electrically heated systems: effect of air and temperatures on the onset of combustion and formation of volatile species. J Anal Appl Pyrolysis 184:106847. 10.1016/j.jaap.2024.106847

[3] U.S. Department of Health and Human Services. How tobacco smoke causes disease: the biology and behavioral basis for smoking-attributable disease: a report of the Surgeon General. 2010, U.S. Department of Health and Human Services, Centers for Disease Control and Prevention, National Center for Chronic Disease Prevention and Health Promotion, Office on Smoking and Health ISBN: 978-0-16-084078-4 21452462

[4] Mallock N, Pieper E, Hutzler C, Henkler-Stephani F, Luch A (2019) Heated tobacco products: a review of current knowledge and initial assessments. Front Public Health 7:287. 10.3389/fpubh.2019.00287 31649912 10.3389/fpubh.2019.00287 PMC6795920

[5] Takahashi Y, Matsumura K, Fukudomi H, Bach R, Hirabayashi T, Sato S, Horiuchi N, Azegami Y (2025) Confirmation of absence of combustion in an electronically heated tobacco product using multiple methods. Next Res 2(2):100294. 10.1016/j.nexres.2025.100294

[6] Cozzani V, Barontini F, McGrath T, Mahler B, Nordlund M, Smith M, Schaller JP, Zuber G (2020) An experimental investigation into the operation of an electrically heated tobacco system. Thermochim Acta 684:178475. 10.1016/j.tca.2019.178475

[7] Hashizume T, Ishikawa S, Matsumura K, Ito S, Fukushima T (2023) Chemical and in vitro toxicological comparison of emissions from a heated tobacco product and the 1R6F reference cigarette. Toxicol Rep 10:281–292. 10.1016/j.toxrep.2023.02.005 36876026 10.1016/j.toxrep.2023.02.005 PMC9976195

[8] Schaller JP, Keller D, Poget L, Pratte P, Kaelin E, McHugh D, Cudazzo G, Smart D, Tricker AR, Gautier L, Yerly M, Reis Pires R, Le Bouhellec S, Ghosh D, Hofer I, Garcia E, Vanscheeuwijck P, Maeder S (2016) Evaluation of the Tobacco Heating System 2.2. Part 2: chemical composition, genotoxicity, cytotoxicity, and physical properties of the aerosol. Regul Toxicol Pharmacol 81(Suppl 2):S27–S47. 10.1016/j.yrtph.2016.10.001 27720919 10.1016/j.yrtph.2016.10.001

[9] Forster M, Fiebelkorn S, Yurteri C, Mariner D, Liu C, Wright C, McAdam K, Murphy J, Proctor C (2018) Assessment of novel tobacco heating product THP1.0. Part 3: comprehensive chemical characterisation of harmful and potentially harmful aerosol emissions. Regul Toxicol Pharmacol 93:14–33. 10.1016/j.yrtph.2017.10.006 29080848 10.1016/j.yrtph.2017.10.006

[10] Nishihara D, Yuki D, Suzuki T, Sakaguchi C, Nagata Y, Kakehi A (2024) A randomized control study in healthy adult smokers to assess reduced exposure to selected cigarette smoke constituents in switching to the novel heated tobacco product DT3.0a. Clin Pharmacol Drug Dev 13(1):45–57. 10.1002/cpdd.1322 37680118 10.1002/cpdd.1322

[11] Haziza C, de La Bourdonnaye G, Merlet S, Benzimra M, Ancerewicz J, Donelli A, Baker G, Picavet P, Ludicke F (2016) Assessment of the reduction in levels of exposure to harmful and potentially harmful constituents in Japanese subjects using a novel tobacco heating system compared with conventional cigarettes and smoking abstinence: a randomized controlled study in confinement. Regul Toxicol Pharmacol 81:489–499. 10.1016/j.yrtph.2016.09.014 27693654 10.1016/j.yrtph.2016.09.014

[12] Haziza C, de La Bourdonnaye G, Donelli A, Poux V, Skiada D, Weitkunat R, Baker G, Picavet P, Ludicke F (2020) Reduction in exposure to selected harmful and potentially harmful constituents approaching those observed upon smoking abstinence in smokers switching to the Menthol Tobacco Heating System 2.2 for 3 months (Part 1). Nicotine Tob Res 22(4):539–548. 10.1093/ntr/ntz013 30722062 10.1093/ntr/ntz013 PMC7164581

[13] Ludicke F, Picavet P, Baker G, Haziza C, Poux V, Lama N, Weitkunat R (2018) Effects of switching to the Tobacco Heating System 2.2 Menthol, smoking abstinence, or continued cigarette smoking on biomarkers of exposure: a randomized, controlled, open-label, multicenter study in sequential confinement and ambulatory settings (Part 1). Nicotine Tob Res 20(2):161–172. 10.1093/ntr/ntw287 28177489 10.1093/ntr/ntw287 PMC5896533

[14] Gale N, McEwan M, Eldridge AC, Fearon IM, Sherwood N, Bowen E, McDermott S, Holmes E, Hedge A, Hossack S, Wakenshaw L, Glew J, Camacho OM, Errington G, McAughey J, Murphy J, Liu C, Proctor CJ (2019) Changes in biomarkers of exposure on switching from a conventional cigarette to tobacco heating products: a randomized, controlled study in healthy Japanese subjects. Nicotine Tob Res 21(9):1220–1227. 10.1093/ntr/nty104 29912406 10.1093/ntr/nty104 PMC6698948

[15] Gale N, McEwan M, Hardie G, Proctor CJ, Murphy J (2022) Changes in biomarkers of exposure and biomarkers of potential harm after 360 days in smokers who either continue to smoke, switch to a tobacco heating product or quit smoking. Intern Emerg Med 17(7):2017–2030. 10.1007/s11739-022-03062-1 36036342 10.1007/s11739-022-03062-1 PMC9522838

[16] Dempsey R, Gunduz I, Vanscheeuwijck P (2025) Bridging approaches to facilitate innovation: building an approach for heated tobacco products from case studies in the food and drug domains a comparative review. Arch Toxicol. 10.1007/s00204-025-04081-5 40399495 10.1007/s00204-025-04081-5 PMC12367943

[17] Goodall S, Gale N, Thorne D, Hadley S, Prasad K, Gilmour I, Miazzi F, Proctor C (2022) Evaluation of behavioural, chemical, toxicological and clinical studies of a tobacco heated product glo and the potential for bridging from a foundational dataset to new product iterations. Toxicol Rep 9:1426–1442. 10.1016/j.toxrep.2022.06.014 36561950 10.1016/j.toxrep.2022.06.014 PMC9764197

[18] Maeder S, Jeannet C (2025) Comparative assessment of the FDA list of 93 HPHCs in aerosol generated by tobacco heating system 2.2 versus 3R4F reference cigarette smoke. Chem Res Toxicol 38(6):1037–1045. 10.1021/acs.chemrestox.4c00544 40396687 10.1021/acs.chemrestox.4c00544 PMC12175160

[19] Jaccard G, Tafin Djoko D, Moennikes O, Jeannet C, Kondylis A, Belushkin M (2017) Comparative assessment of HPHC yields in the Tobacco Heating System THS2.2 and commercial cigarettes. Regul Toxicol Pharmacol 90:1–8. 10.1016/j.yrtph.2017.08.006 28818540 10.1016/j.yrtph.2017.08.006

[20] Gunduz I, Nordlund M, King J, Gustin B, Cudazzo G, Nesovic M, Butin Y, Stura E, Alriquet M, Chauhan M, Rossoll A, Szostak J, Belushkin M (2025) A comparative assessment of HPHC yields and in vitro toxicity for 1R6F reference cigarette smoke versus aerosol generated by Tobacco Heating System 3.0. Aerosol Sci Technol 59(2):146–162. 10.1080/02786826.2024.2403573

[21] International Organization for Standardization (2023) ISO 3402:2023 Tobacco and tobacco products – atmosphere for conditioning and testing. https://www.iso.org/standard/83089.html

[22] International Organization for Standardization (2018) ISO 20778:2018 Cigarettes – routine analytical cigarette smoking machine – definitions and standard conditions with an intense smoking regime. https://www.iso.org/standard/69065.html

[23] International Organization for Standardization (2024) ISO 5501-1:2024 Tobacco heating systems – definitions and standard conditions for aerosol generation and collection. Part 1: electrically heated tobacco products. https://www.iso.org/standard/85388.html

[24] World Health Organization (2015) Report on the scientific basis of tobacco product regulation: fifth report of a WHO study group, WHO Technical Report Series No. 989, ISBN 978 92 4 120989 2. https://iris.who.int/bitstream/handle/10665/161512/9789241209892.pdf?sequence=1 26353746

[25] U.S. Department of Health and Human Services, Food and Drug Administration (2012) Reporting harmful and potentially harmful constituents in tobacco products and tobacco smoke under section 904(a)(3) of the Federal Food, Drug, and Cosmetic Act. Draft guidance for industry. https://www.fda.gov/media/83375/download

[26] Health Canada (2019) Tobacco Reporting Regulations, SOR/2000-273. https://laws-lois.justice.gc.ca/PDF/SOR-2000-273.pdf

[27] Yuki D, Takeshige Y, Nakaya K, Futamura Y (2018) Assessment of the exposure to harmful and potentially harmful constituents in healthy Japanese smokers using a novel tobacco vapor product compared with conventional cigarettes and smoking abstinence. Regul Toxicol Pharmacol 96:127–134. 10.1016/j.yrtph.2018.05.001 29738810 10.1016/j.yrtph.2018.05.001

[28] Levy DD, Zeiger E, Escobar PA, Hakura A, van der Leede BM, Kato M, Moore MM, Sugiyama KI (2019) Recommended criteria for the evaluation of bacterial mutagenicity data (Ames test). Mutat Res Genet Toxicol Environ Mutagen 848:403074. 10.1016/j.mrgentox.2019.07.004 31708073 10.1016/j.mrgentox.2019.07.004

[29] Thompson M (2000) Recent trends in inter-laboratory precision at ppb and sub-ppb concentrations in relation to fitness for purpose criteria in proficiency testing. Analyst 125(3):385–386. 10.1039/B000282H

[30] Akiyama Y, Sherwood N (2021) Systematic review of biomarker findings from clinical studies of electronic cigarettes and heated tobacco products. Toxicol Rep 8:282–294. 10.1016/j.toxrep.2021.01.014 33552927 10.1016/j.toxrep.2021.01.014 PMC7850959

[31] Yuki D, Kikuchi A, Suzuki T, Sakaguchi C, Huangfu D, Nagata Y, Kakehi A (2022) Assessment of the exposure to selected smoke constituents in adult smokers using in-market heated tobacco products: a randomized, controlled study. Sci Rep 12(1):18167. 10.1038/s41598-022-22997-1 36307514 10.1038/s41598-022-22997-1 PMC9616951

[32] Bosilkovska M, Tran CT, de La Bourdonnaye G, Taranu B, Benzimra M, Haziza C (2020) Exposure to harmful and potentially harmful constituents decreased in smokers switching to carbon-heated tobacco product. Toxicol Lett 330:30–40. 10.1016/j.toxlet.2020.04.013 32380119 10.1016/j.toxlet.2020.04.013

[33] Ludicke F, Haziza C, Weitkunat R, Magnette J (2016) Evaluation of biomarkers of exposure in smokers switching to a carbon-heated tobacco product: a controlled, randomized, open-label 5-day exposure study. Nicotine Tob Res 18(7):1606–1613. 10.1093/ntr/ntw022 26817490 10.1093/ntr/ntw022 PMC4902889

[34] Haziza C, de La Bourdonnaye G, Skiada D, Ancerewicz J, Baker G, Picavet P, Ludicke F (2017) Biomarker of exposure level data set in smokers switching from conventional cigarettes to Tobacco Heating System 2.2, continuing smoking or abstaining from smoking for 5 days. Data Brief 10:283–293. 10.1016/j.dib.2016.11.047 27995164 10.1016/j.dib.2016.11.047 PMC5156600

[35] Ludicke F, Baker G, Magnette J, Picavet P, Weitkunat R (2017) Reduced exposure to harmful and potentially harmful smoke constituents with the tobacco heating system 2.1. Nicotine Tob Res 19(2):168–175. 10.1093/ntr/ntw164 27613951 10.1093/ntr/ntw164 PMC5234364

[36] Tricker AR, Kanada S, Takada K, Martin Leroy C, Lindner D, Schorp MK, Dempsey R (2012) Reduced exposure evaluation of an Electrically Heated Cigarette Smoking System. Part 6: 6-day randomized clinical trial of a menthol cigarette in Japan. Regul Toxicol Pharmacol 64(2 Suppl):S64–S73. 10.1016/j.yrtph.2012.08.007 22951347 10.1016/j.yrtph.2012.08.007

[37] Frost-Pineda K, Zedler BK, Oliveri D, Feng S, Liang Q, Roethig HJ (2008) Short-term clinical exposure evaluation of a third-generation electrically heated cigarette smoking system (EHCSS) in adult smokers. Regul Toxicol Pharmacol 52(2):104–110. 10.1016/j.yrtph.2008.05.016 18640172 10.1016/j.yrtph.2008.05.016

[38] Tran CT, Bosilkovska M, de La Bourdonnaye G, Blanc N, Haziza C (2020) Reduced levels of biomarkers of exposure in smokers switching to the Carbon-Heated Tobacco Product 1.0: a controlled, randomized, open-label 5-day exposure trial. Sci Rep 10(1):19227. 10.1038/s41598-020-76222-y 33154508 10.1038/s41598-020-76222-y PMC7644773

[39] Tricker AR, Kanada S, Takada K, Leroy CM, Lindner D, Schorp MK, Dempsey R (2012) Reduced exposure evaluation of an Electrically Heated Cigarette Smoking System. Part 5: 8-day randomized clinical trial in Japan. Regul Toxicol Pharmacol 64(2 Suppl):S54–S63. 10.1016/j.yrtph.2012.08.003 22940437 10.1016/j.yrtph.2012.08.003

[40] Martin Leroy C, Jarus-Dziedzic K, Ancerewicz J, Lindner D, Kulesza A, Magnette J (2012) Reduced exposure evaluation of an Electrically Heated Cigarette Smoking System. Part 7: a one-month, randomized, ambulatory, controlled clinical study in Poland. Regul Toxicol Pharmacol 64(2 Suppl):S74–S84. 10.1016/j.yrtph.2012.08.006 22951349 10.1016/j.yrtph.2012.08.006

[41] Tricker AR, Jang IJ, Martin Leroy C, Lindner D, Dempsey R (2012) Reduced exposure evaluation of an Electrically Heated Cigarette Smoking System. Part 4: eight-day randomized clinical trial in Korea. Regul Toxicol Pharmacol 64(2 Suppl):S45–S53. 10.1016/j.yrtph.2012.08.013 22951346 10.1016/j.yrtph.2012.08.013

[42] Sakaguchi C, Kakehi A, Minami N, Kikuchi A, Futamura Y (2014) Exposure evaluation of adult male Japanese smokers switched to a heated cigarette in a controlled clinical setting. Regul Toxicol Pharmacol 69(3):338–347. 10.1016/j.yrtph.2014.04.016 24819671 10.1016/j.yrtph.2014.04.016

[43] Ludicke F, Ansari SM, Lama N, Blanc N, Bosilkovska M, Donelli A, Picavet P, Baker G, Haziza C, Peitsch M, Weitkunat R (2019) Effects of switching to a heat-not-burn tobacco product on biologically relevant biomarkers to assess a candidate modified risk tobacco product: a randomized trial. Cancer Epidemiol Biomarkers Prev 28(11):1934–1943. 10.1158/1055-9965.EPI-18-0915 31270101 10.1158/1055-9965.EPI-18-0915

[44] Shepperd CJ, Newland N, Eldridge A, Haswell L, Lowe F, Papadopoulou E, Camacho O, Proctor CJ, Graff D, Meyer I (2015) Changes in levels of biomarkers of exposure and biological effect in a controlled study of smokers switched from conventional cigarettes to reduced-toxicant-prototype cigarettes. Regul Toxicol Pharmacol 72(2):273–291. 10.1016/j.yrtph.2015.04.016 25957570 10.1016/j.yrtph.2015.04.016

[45] Ogden MW, Marano KM, Jones BA, Morgan WT, Stiles MF (2015) Switching from usual brand cigarettes to a tobacco-heating cigarette or snus: part 2. Biomarkers of exposure. Biomarkers 20(6–7):391–403. 10.3109/1354750X.2015.1094134 26554277 10.3109/1354750X.2015.1094134 PMC4720046

[46] Roethig HJ, Feng S, Liang Q, Liu J, Rees WA, Zedler BK (2008) A 12-month, randomized, controlled study to evaluate exposure and cardiovascular risk factors in adult smokers switching from conventional cigarettes to a second-generation electrically heated cigarette smoking system. J Clin Pharmacol 48(5):580–591. 10.1177/0091270008315316 18319361 10.1177/0091270008315316

[47] Tricker AR, Stewart AJ, Leroy CM, Lindner D, Schorp MK, Dempsey R (2012) Reduced exposure evaluation of an Electrically Heated Cigarette Smoking System. Part 3: eight-day randomized clinical trial in the UK. Regul Toxicol Pharmacol 64(2 Suppl):S35–S44. 10.1016/j.yrtph.2012.08.010 22940436 10.1016/j.yrtph.2012.08.010

[48] Frost-Pineda K, Zedler BK, Oliveri D, Liang Q, Feng S, Roethig HJ (2008) 12-week clinical exposure evaluation of a third-generation electrically heated cigarette smoking system (EHCSS) in adult smokers. Regul Toxicol Pharmacol 52(2):111–117. 10.1016/j.yrtph.2008.05.015 18619511 10.1016/j.yrtph.2008.05.015

[49] European Chemical Agency. Guide for enforcement of mixture classification based on bridging principles. Article 9(4) of the CLP Regulation Weight of evidence/Expert judgements, July 2024, update in September 2024, ECHA-24-H-10-EN, ISBN: 978-92-9468-390-8, Cat. Number: ED-09-24-605-EN-N. 10.2823/475106

[50] Medicines and Healthcare products Regulatory Agency. Guidance Chapter 3 – emissions from electronic cigarettes – GB, 2022, last updated 16 August 2024. https://www.gov.uk/government/publications/chapter-3-emissions-guidance-great-britain/chapter-3-emissions-from-electronic-cigarettes-gb

