# Supplementary material for: Assessing the comparability of toxic emissions reduction from heated tobacco aerosols relative to cigarette smoke: a scientific approach to bridging datasets
Source: Intern Emerg Med. 2025 Oct 29;21(1):83–99. doi: 10.1007/s11739-025-04160-6 (PMC12948778; doi:10.1007/s11739-025-04160-6)
Supplement: Supplementary file 1 — Supplementary file1 (DOCX 2737 KB) [file 11739_2025_4160_MOESM1_ESM.docx]

**Supplementary** **Materials**

**Assessing the comparability of toxic emissions reduction from heated tobacco aerosols relative to cigarette smoke: a scientific approach to bridging datasets**

**J. MILLER-HOLT^1^*,** **G. O’CONNELL^1^, R. BACH^1^,** **M. CHARRIERE^1^,** **Y. KANEMARU^1^, Z. SU^2^, S. LARROQUE^1^, K. JACOBSON^1^**

*Author to whom correspondence should be addressed.

JTI SA, 8 rue Kazem Radjavi, 1202, Geneva, Switzerland^1^

Japan Tobacco Inc., 6-2, Umegaoka, Aoba-ku, Yokohama, Kanagawa, 227-8512, Japan^2^

**KEYWORDS.** Emissions, Statistics, Heated Tobacco, Bridging, Biomarker of Exposure, Harm Reduction.

**ORCID iD**: J. MILLER-HOLT (0000-0001-5815-0552), G. O’CONNELL (0009-0002-8877-1629), R. BACH (0009-0007-4913-1262), M. CHARRIERE (0000-0003-1668-8375), Y. KANEMARU (0000-0001-9934-9488), Z. SU (0000-0002-7987-4442), K. JACOBSON (0000-0002-4673-1592).

Supplementary Table S1: Description of the standardized puffing regimes used to generate the aerosol for heated tobacco sticks and the smoke of cigarette

| Variable | ISO 5501-1: 2024 | ISO 20778: 2018 |
| --- | --- | --- |
| Puff Volume (mL) | 55 | 55 |
| Puff Interval (sec) | 30 | 30 |
| Puff Duration (sec) | 2 | 2 |
| Puff Profile | Bell Shape | Bell Shape |
| Vent Blocking | Open | Closed |
|  | |  |

Supplementary Table S2: List of analytes evaluated in this study and summary of the methods applied in the accredited laboratories for their respective quantification in the aerosol from HTS

| Analyte | Analytical Method | Method Description |
| --- | --- | --- |
| Ammonia | TMS-00101 Appendix E  Or ISO 23920 | Ammonia in Mainstream Heat-Not-Burn Emissions by Ion Chromatography (EC) |
| Formaldehyde  Acetaldehyde  Acetone  Acrolein  Propionaldehyde  Crotonaldehyde  Methyl Ethyl Ketone  Butyraldehyde | TMS-00104  Or ISO 23922 | Carbonyls in Mainstream Heat-Not-Burn Emissions by HPLC-UV |
| Hydrogen cyanide (HCN) | TMS-00107 Appendix C | Hydrogen Cyanide (HCN) in Mainstream Heat-Not-Burn Emissions by Continuous Flow Analysis |
| Mercury | TMS-00108 Appendix F | Mercury in Mainstream Heat-not-Burn Emissions by Cold Vapor Atomic Absorption Spectroscopy, |
| Cadmium  Lead  Chromium  Nickel  Arsenic  Selenium | TMS-00109 Appendix H | Metals in Mainstream Heat-not-Burn Emissions by ICP-MS using ultrapure reagents |
| Nitric Oxide (NO)  Nitrogen Oxides (NOx) | TMS-00110 Appendix H | Nitrogen Oxides in Mainstream Heat-Not-Burn Emissions using a Chemiluminescence Analyzer |
| Pyridine  Quinoline  Styrene  Nitrobenzene  Acetamide  Acrylamide | TMS-00112 Appendix G  Or ISO 23923 | Semi-Volatile Compounds in Mainstream Heat-Not-Burn Emissions by GC-MS (SIM) |
| Carbon Monoxide (CO)  Nicotine-Free Dry Particulate Matter (NFDPM) | TMS-00115a Appendix E  Or ISO 22253/ ISO 20779 and ISO 22947 | Nicotine, Carbon Monoxide, Menthol and Humectants in Mainstrean Heat-Not-Burn Emissions by GC-TCD/FID |
| Pyrene  Benzo(a)anthracene  Benzo(a)pyrene  Dibenz(a,h)anthracene | TMS-00120 Appendix H  Or ISO 23906-1 | Polynuclear Aromatic Hydrocarbons in Mainstream Heat-Not-Burn Emissions by GC-MS (SIM) |
| 1,3-butadiene  Isoprene  Acrylonitrile  Benzene  Toluene  Ethylene Oxide  Vinyl Chloride  Propylene Oxide | TMS-00124 Appendix G  Or ISO 23923 | Volatile Compounds in Mainstream Heat-Not-Burn Emissions by GC-MS (SIM) |
| 1-aminonaphthalene  2-aminonaphthalene  3-aminobiphenyl  4-aminobiphenyl  o-toluidine | TMS-00128 Appendix G  Or T-102 | Aromatic Amines in Mainstream Heat-Not-Burn Emissions by GC-MS (NCI) |
| N-nitrosonornicotine (NNN)  4-(N-nitrosomethylamino)-1-(3-pyridyl)-1-butanone (NNK)  N-nitrosoanatabine (NAT)  N-nitrosoanabasine (NAB) | TMS-00135  or ISO 23921 | Tobacco Specific Nitrosamines in Mainstream Heat-Not-Burn Emissions by LC-MS/MS |
| Hydroquinone  Resorcinol  Catechol  Phenol  m-Cresol  p-Cresol  o-Cresol | TMS-00139  Appendix F  Or ISO 23904 | Phenols In Mainstream Heat-Not-Burn Emissions by HPLC-FLU |

Supplementary Table S3: Summary of measured mass median aerodynamic diameter and geometric standard deviation of 1R6F cigarette smoke, HTS aerosol generated with device A and B and commercial HTS comparator aerosol (n=6)

| **Device** | **MMAD (mean) [µm]** | **GSD (mean)** |
| --- | --- | --- |
| HTS Device A | 0.66 | 1.56 |
| HTS Device B | 0.69 | 1.53 |
| Commercial HTS Comparator | 0.45 | 1.54 |
| 1R6F Combustible Cigarette Smoke | 0.72 | 1.41 |

MMAD: mass median aerodynamic diameter, micrometres (µm); GSD geometric, standard deviation of the mass aerosol distribution.

Supplementary Table S4: Summary of the mutagenicity evaluation of individual HTS test items and 1R6F cigarette in Ames assay

| **Test item ID** | **Stick type** | **Device type** | **Particulate phase (TPM or ACM)** | | | | | | | | | | **Gas vapor phase (GVP)** | | | | | | | | | |
| --- | --- | --- | --- | --- | --- | --- | --- | --- | --- | --- | --- | --- | --- | --- | --- | --- | --- | --- | --- | --- | --- | --- |
|  |  |  | **TA98 + S9** | **TA100 + S9** | **TA102 + S9** | **TA1535 + S9** | **TA1537 + S9** | **TA98 - S9** | **TA100 - S9** | **TA102 - S9** | **TA1535 - S9** | **TA1537 - S9** | **TA98 + S9** | **TA100 + S9** | **TA102 + S9** | **TA1535 + S9** | **TA1537 + S9** | **TA98 - S9** | **TA100 - S9** | **TA102 - S9** | **TA1535 - S9** | **TA1537 - S9** |
| 1804571 | Regular | Device A | - | - | - | - | - | - | - | - | - | - | - | - | - | - | - | - | - | - | - | - |
| 1804574 | Regular | Device A | - | - | - | - | - | - | - | - | - | - | - | - | - | - | - | - | - | - | - | - |
| 1821138 | Regular | Device A | - | - | - | - | - | - | - | - | - | - | - | - | - | - | - | - | - | - | - | - |
| 1821139 | Regular | Device A | - | - | - | - | - | - | - | - | - | - | - | - | - | - | - | - | - | - | - | - |
| 1876668 | Regular | Device A | - | - | - | - | - | - | - | - | - | - | - | - | - | - | - | - | - | - | - | - |
| 1876669 | Regular | Device A | - | - | - | - | - | - | - | - | - | - | - | - | - | - | - | - | - | - | - | - |
| 1869726 | Regular | Device A | - | - | - | - | - | - | - | - | - | - | - | - | - | - | - | - | - | - | - | - |
| 1876665 | Regular | Device A | - | - | - | - | - | - | - | - | - | - | - | - | - | - | - | - | - | - | - | - |
| 1906141 | Non-menthol | Device A | - | - | - | - | - | - | - | - | - | - | - | - | - | - | - | - | - | - | - | - |
| 1906142 | Non-menthol | Device A | - | - | - | - | - | - | - | - | - | - | - | - | - | - | - | - | - | - | - | - |
| 1906143 | Non-menthol | Device A | - | - | - | - | - | - | - | - | - | - | - | - | - | - | - | - | - | - | - | - |
| 1906144 | Non-menthol | Device A | - | - | - | - | - | - | - | - | - | - | - | - | - | - | - | - | - | - | - | - |
| 1821574 | Menthol | Device A | - | - | - | - | - | - | - | - | - | - | - | - | - | - | - | - | - | - | - | - |
| 1879014 | Menthol | Device A | - | - | - | - | - | - | - | - | - | - | - | - | - | - | - | - | - | - | - | - |
| 1870454 | Menthol | Device A | - | - | - | - | - | - | - | - | - | - | - | - | - | - | - | - | - | - | - | - |
| 1906145 | Menthol | Device A | - | - | - | - | - | - | - | - | - | - | - | - | - | - | - | - | - | - | - | - |
| 1906146 | Menthol | Device A | - | - | - | - | - | - | - | - | - | - | - | - | - | - | - | - | - | - | - | - |
| 1917227-1923945 | Regular | Device B | - | - | - | - | - | - | - | - | - | - | - | - | - | - | - | - | - | - | - | - |
| 1917228-1923946 | Regular | Device B | - | - | - | - | - | - | - | - | - | - | - | - | - | - | - | - | - | - | - | - |
| 1917229-1923947 | Regular | Device B | - | - | - | - | - | - | - | - | - | - | - | - | - | - | - | - | - | - | - | - |
| 1917230-1923948 | Regular | Device B | - | - | - | - | - | - | - | - | - | - | - | - | - | - | - | - | - | - | - | - |
| 1917231-1923949 | Regular | Device B | - | - | - | - | - | - | - | - | - | - | - | - | - | - | - | - | - | - | - | - |
| 1917232-1923950 | Regular | Device B | - | - | - | - | - | - | - | - | - | - | - | - | - | - | - | - | - | - | - | - |
| 1895660 | 1R6F | N/A | + (1021.0) | -* | - | - | -* | -* | -* | - | - | - | - | - | - | - | - | - | - | - | - | - |
| 1905112 | 1R6F | N/A | + (1517.6) | -* | - | - | + (217.2) | -* | -* | - | - | - | - | - | - | - | - | - | - | - | - | - |
| 1906955 | 1R6F | N/A | + (1359.3) | -* | - | - | + (216.4) | -* | -* | - | - | -* | - | - | - | - | - | - | - | - | - | - |
| 1922872 | 1R6F | N/A | + (1414.3) | -* | - | - | -* | -* | -* | - | - | -* | - | - | - | - | - | - | - | - | - | - |

"N/A" denotes not applicable. "+S9" denotes in the presence of metabolic activation. "-S9" denotes in the absence of metabolic activation. "+" denotes positive (mutagenic) response in the particular strain and metabolic activation condition. "-" denotes negative (non-mutagenic) response in the particular strain and metabolic activation condition. "-*" denotes negative response accompanied by a concentration-related increase in the number of revertants, although the highest number of revertants did not meet the criteria for mutagenicity evaluation in the particular strain and metabolic activation condition. The value in parentheses is the mean mutagenicity slope in the number of revertants (per plate)/mg TPM unit, estimated from the linear portion of the dose-response curve.

Supplementary Table S5: Summary of mutagenic responses in the particulate and gas-vapor phases of HTS aerosol and 1R6F cigarette smoke

| Test | Tester | Metabolic | HTS | | | | | |  | Cigarette |
| --- | --- | --- | --- | --- | --- | --- | --- | --- | --- | --- |
| fraction | strain | activation | Per stick type | | |  | Per device type | |  |  |
|  |  |  | Regular | Non-menthol | Menthol |  | Device A | Device B |  | 1R6F |
| PP | TA98 | -S9 | - | - | - |  | - | - |  | - |
|  |  | +S9 | - | - | - |  | - | - |  | + |
|  | TA100 | -S9 | - | - | - |  | - | - |  | - |
|  |  | +S9 | - | - | - |  | - | - |  | - |
|  | TA102 | -S9 | - | - | - |  | - | - |  | - |
|  |  | +S9 | - | - | - |  | - | - |  | - |
|  | TA1535 | -S9 | - | - | - |  | - | - |  | - |
|  |  | +S9 | - | - | - |  | - | - |  | - |
|  | TA1537 | -S9 | - | - | - |  | - | - |  | - |
|  |  | +S9 | - | - | - |  | - | - |  | + |
|  |  |  |  |  |  |  |  |  |  |  |
| GVP | TA98 | -S9 | - | - | - |  | - | - |  | - |
|  |  | +S9 | - | - | - |  | - | - |  | - |
|  | TA100 | -S9 | - | - | - |  | - | - |  | - |
|  |  | +S9 | - | - | - |  | - | - |  | - |
|  | TA102 | -S9 | - | - | - |  | - | - |  | - |
|  |  | +S9 | - | - | - |  | - | - |  | - |
|  | TA1535 | -S9 | - | - | - |  | - | - |  | - |
|  |  | +S9 | - | - | - |  | - | - |  | - |
|  | TA1537 | -S9 | - | - | - |  | - | - |  | - |
|  |  | +S9 | - | - | - |  | - | - |  | - |

Abbreviations: PP=particulate phase, GVP=gas vapor phase; HTS=heated tobacco stick; -=non-mutagenic response observed; +=mutagenic response observed. Number of datasets: HTS Regular=14; HTS Non-menthol=4; HTS Menthol=5; HTS Device A=17; HTS Device B=6; 1R6F = 4. Three independent replicates per test sample.

Supplementary Table S6: Summary of the genotoxicity evaluation of individual HTS test items and 1R6F cigarette in *in vitro* micronucleus assay

| **Test item ID** | **Stick type** | **Device type** | **Particulate phase (TPM or ACM)** | | | **Gas vapor phase (GVP)** | | |
| --- | --- | --- | --- | --- | --- | --- | --- | --- |
|  |  |  | **Short (3 h) - S9** | **Short (3 h) + S9** | **Long (30 h) - S9** | **Short (3 h) - S9** | **Short (3h) + S9** | **Long (30 h) - S9** |
| 1804571 | Regular | Device A | + (2.774) | - (2.301) | + (4.095) | - (1.649) | - (2.160) | - (2.483) |
| 1804574 | Regular | Device A | - (2.610) | - (1.771) | + (5.785) | - (2.197) | - (1.943) | - (3.473) |
| 1821138 | Regular | Device A | - (2.774) | - (1.460) | - (4.097) | + (2.258) | - (1.640) | + (5.292) |
| 1821139 | Regular | Device A | + (2.520) | - (1.135) | + (4.339) | - (1.738) | - (0.868) | - (2.621) |
| 1876668 | Regular | Device A | - (1.861) | - (1.923) | - (3.599) | + (2.362) | + (2.612) | - (3.199) |
| 1876669 | Regular | Device A | + (3.055) | + (1.960) | - (3.234) | - (1.041) | - (1.442) | + (4.110) |
| 1869726 | Regular | Device A | - (2.311) | - (1.352) | + (5.977) | + (2.151) | - (1.562) | - (3.086) |
| 1876665 | Regular | Device A | - (1.699) | - (1.152) | - (2.417) | - (1.971) | - (1.535) | - (2.571) |
| 1906141 | Non-menthol | Device A | + (2.048) | - (1.758) | + (4.343) | + (2.424) | + (2.115) | + (5.140) |
| 1906142 | Non-menthol | Device A | + (2.382) | - (2.003) | + (5.005) | - (2.153) | - (1.528) | - (4.099) |
| 1906143 | Non-menthol | Device A | + (2.244) | - (2.116) | - (4.635) | - (2.357) | - (2.076) | + (4.987) |
| 1906144 | Non-menthol | Device A | + (2.656) | - (1.958) | - (4.011) | - (1.744) | - (1.800) | - (4.290) |
| 1821574 | Menthol | Device A | - (1.994) | + (3.709) | - (1.922) | - (1.490) | - (2.746) | - (2.240) |
| 1879014 | Menthol | Device A | - (1.958) | + (2.374) | - (3.158) | - (1.365) | + (2.708) | - (4.213) |
| 1870454 | Menthol | Device A | - (2.000) | - (2.894) | + (4.865) | + (2.355) | - (1.338) | - (2.934) |
| 1906145 | Menthol | Device A | + (2.724) | - (2.470) | - (3.879) | + (2.197) | - (2.134) | - (3.597) |
| 1906146 | Menthol | Device A | + (2.624) | - (2.449) | + (4.778) | - (2.183) | - (1.351) | - (4.204) |
| 1917227-1923945 | Regular | Device B | + (1.873) | + (2.193) | + (3.159) | + (2.216) | - (2.026) | + (3.678) |
| 1917228-1923946 | Regular | Device B | + (1.790) | + (2.315) | + (5.997) | + (2.467) | + (3.129) | + (3.978) |
| 1917229-1923947 | Regular | Device B | + (2.301) | + (2.419) | + (4.263) | + (2.293) | + (2.194) | + (4.249) |
| 1917230-1923948 | Regular | Device B | + (2.089) | + (2.723) | + (2.173) | + (2.213) | + (3.008) | + (4.653) |
| 1917231-1923949 | Regular | Device B | + (1.781) | + (2.647) | + (3.582) | + (2.380) | + (3.072) | - (3.803) |
| 1917232-1923950 | Regular | Device B | + (2.183) | + (2.877) | + (2.081) | + (1.904) | + (2.390) | + (3.644) |
| 1895660 | 1R6F | N/A | + (42.799) | + (37.059) | + (93.617) | + (30.723) | + (23.635) | + (52.327) |
| 1905112 | 1R6F | N/A | + (41.016) | + (34.366) | + (111.170) | - (14.825) | + (18.389) | + (30.945) |
| 1906955 | 1R6F | N/A | + (40.706) | + (33.498) | + (82.983) | + (21.548) | + (23.654) | + (45.471) |
| 1922872 | 1R6F | N/A | + (36.980) | + (36.731) | + (69.614) | + (31.504) | + (24.311) | + (47.660) |

"N/A" denotes not applicable."+S9" denotes in presence of metabolic activation. "-S9" denotes in absence of metabolic activation. "+" denotes positive (genotoxic) response in the treatment schedule. "-" denotes negative (non-genotoxic) response in the treatment schedule. The value in parentheses is the mean genotoxicity slope in the number of micronuclei (per 1000 cells)/mg TPM or ACM (equivalent for GVP) /mL unit, estimated from the linear portion of the dose-response curve.


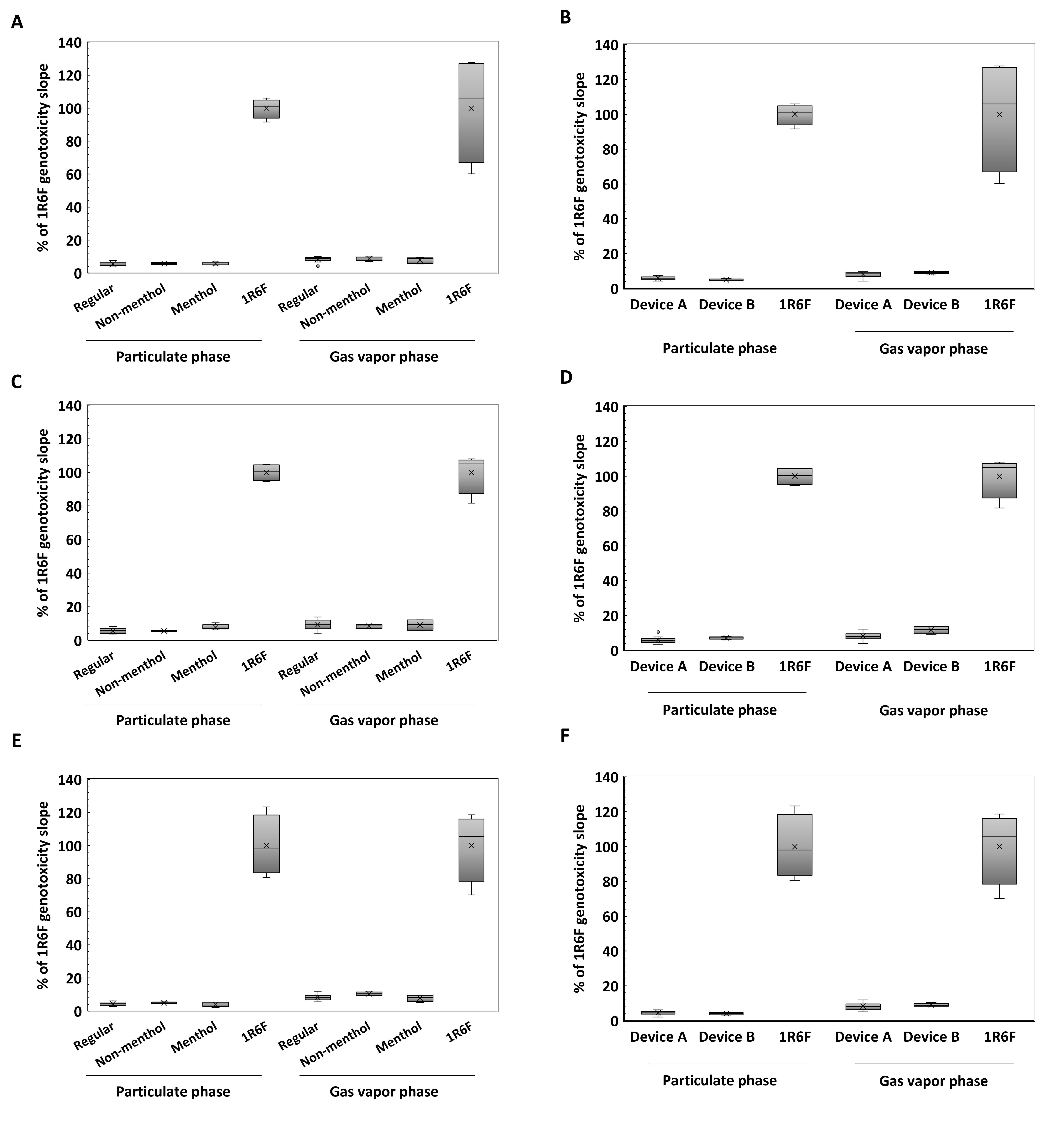


Supplemental Figure S1: Relative comparisons of genotoxic response of HTS aerosols versus 1R6F cigarette smoke on a per extract weight basis

The ivMN assay was performed under three treatment schedules: (A and B) results of short-term (3 h) exposure in absence of metabolic activation, (C and D) results of short-term (3 h) exposure in presence of metabolic activation, and (C and F) results of long-term (30 h) exposure in absence of metabolic activation. The genotoxic responses of HTS test items relative to 1R6F cigarette was calculated using the following formula: genotoxicity slope of HTS test item/mean genotoxicity slope of pooled 1R6F cigarette data × 100. The results of ivMN assay conducted over various time periods were integrated and compared (A, C, and E) per stick type and (B, D, and F) per device type. The box plot extends from the 25th to 75th percentiles, the line in the middle indicates the median, the cross indicates the mean, and the whiskers extend from the smallest and largest values within 1.5 times the interquartile range of the nearer quartile. Number of datasets: HTS Regular=14; HTS Non-menthol=4; HTS Menthol=5; HTS Device A=17; HTS Device B=6; 1R6F=4. Three independent replicates per test sample.

Supplementary Table S7: Summary of IC_50_ values of individual HTS test items and 1R6F cigarette in neutral red uptake assay

| **Test item ID** | **Stick type** | **Device type** | **Particulate phase (TPM or ACM)** | | **Gas vapor phase (GVP)** | |
| --- | --- | --- | --- | --- | --- | --- |
|  |  |  | **Mean IC_50_  (μg ACM or TPM/mL)** | **SD** | **Mean IC_50_  (μg ACM or TPM equivalent/mL)** | **SD** |
| 1804571 | Regular | Device A | 1139.7 | 232.0 | > 2000 | N/A |
| 1804574 | Regular | Device A | 603.4 | 71.3 | 1411.4 | 168.5 |
| 1821138 | Regular | Device A | 693.9 | 79.4 | > 2000 | N/A |
| 1821139 | Regular | Device A | 1081.1 | 110.9 | > 2000 | N/A |
| 1876668 | Regular | Device A | 674.3 | 63.5 | 1716.6 | 139.7 |
| 1876669 | Regular | Device A | 815.1 | 67.3 | > 2000 | N/A |
| 1869726 | Regular | Device A | 1003.3 | 60.6 | > 2000 | N/A |
| 1876665 | Regular | Device A | 999.2 | 42.8 | > 2000 | N/A |
| 1906141 | Non-menthol | Device A | 903.3 | 50.3 | > 2000 | N/A |
| 1906142 | Non-menthol | Device A | 768.2 | 92.8 | > 2000 | N/A |
| 1906143 | Non-menthol | Device A | 791.4 | 6.4 | > 2000 | N/A |
| 1906144 | Non-menthol | Device A | 735.1 | 123.3 | > 2000 | N/A |
| 1821574 | Menthol | Device A | 521.5 | 112.6 | > 2000 | N/A |
| 1879014 | Menthol | Device A | 648.3 | 25.7 | > 2000 | N/A |
| 1870454 | Menthol | Device A | 709.8 | 81.2 | > 2000 | N/A |
| 1906145 | Menthol | Device A | 764.3 | 75.7 | > 2000 | N/A |
| 1906146 | Menthol | Device A | 610.1 | 76.6 | > 2000 | N/A |
| 1917227-1923945 | Regular | Device B | 1184.2 | 291.9 | > 2000 | N/A |
| 1917228-1923946 | Regular | Device B | 998.5 | 116.8 | > 2000 | N/A |
| 1917229-1923947 | Regular | Device B | 859.6 | 249.3 | > 2000 | N/A |
| 1917230-1923948 | Regular | Device B | 847.7 | 173.8 | > 2000 | N/A |
| 1917231-1923949 | Regular | Device B | 1333.6 | 205.2 | > 2000 | N/A |
| 1917232-1923950 | Regular | Device B | 948.4 | 164.7 | > 2000 | N/A |
| 1895660 | 1R6F | N/A | 73.1 | 7.3 | 139.9 | 18.1 |
| 1905112 | 1R6F | N/A | 67.2 | 5.1 | 110.4 | 0.7 |
| 1906955 | 1R6F | N/A | 82.1 | 16.3 | 121.1 | 13.2 |
| 1922872 | 1R6F | N/A | 77.8 | 8.5 | 134.1 | 10.7 |

“SD” denotes standard deviation. "N/A" denotes not applicable. "> 2000" denotes cell viability remaining greater than 50% up to the highest concentration tested (i.e., 2000 µg ACM equivalent/mL). Therefore, the highest concentration was indicated as a conservative assumption.


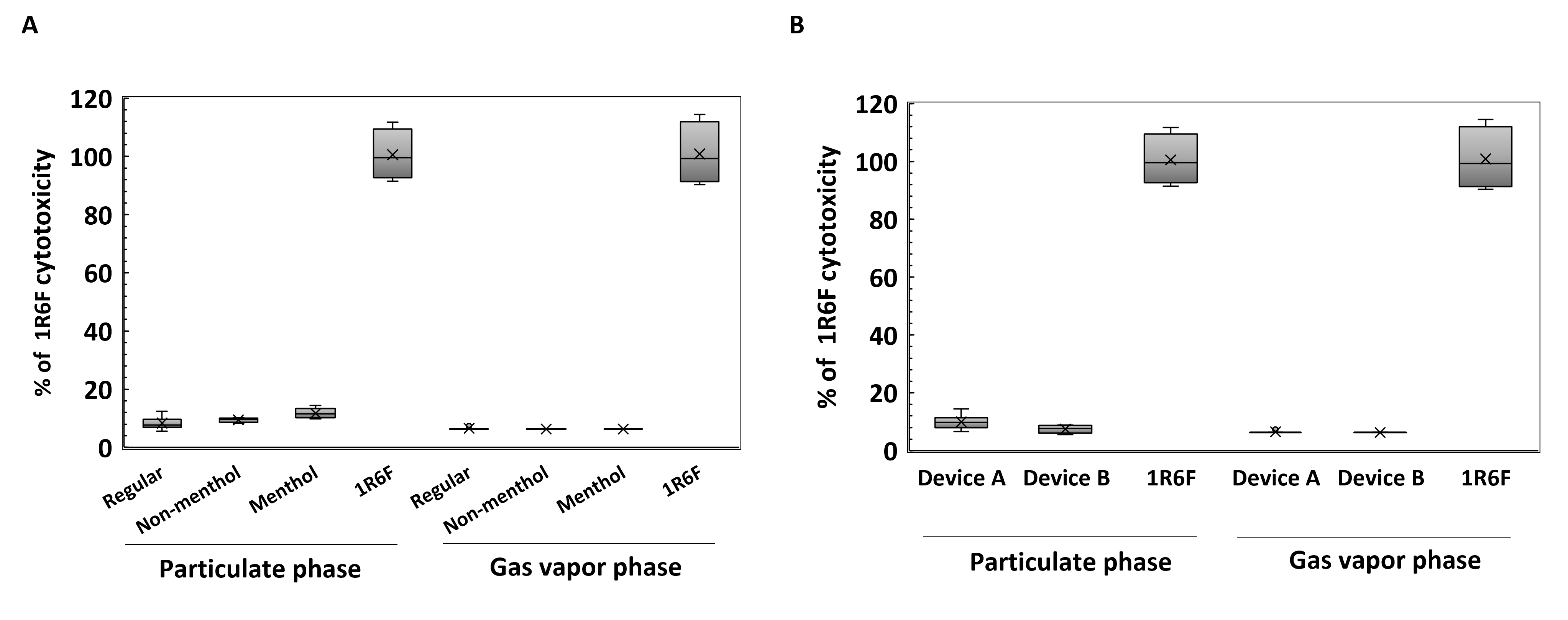


Supplemental Figure S2: Relative comparisons of cytotoxic response of HTS aerosols versus 1R6F cigarette smoke on a per extract weight basis

The cytotoxic responses of HTS test items relative to 1R6F cigarette were calculated using the following formula: (1/IC_50_ value of HTS test item)/(1/mean IC_50_ value of pooled 1R6F cigarette data) × 100. The results of NRU assay over various time periods were integrated and compared (A) per stick type and (B) per device type. The GVP samples from all HTS test items elicited only weak cytotoxic responses, with cell viability remaining above 50% at the highest concentration tested (2000 µg ACM equivalent/mL). Accordingly, the highest concentration was used as a conservative basis for calculating the relative cytotoxicity. The box plot extends from the 25th to 75th percentiles, the line in the middle indicates the median, the cross indicates the mean, and the whiskers extend from the smallest and largest values within 1.5 times the interquartile range of the nearer quartile. Number of datasets: HTS Regular=14; HTS Non-menthol=4; HTS Menthol=5; HTS Device A=17; HTS Device B=6; 1R6F=4. Three independent replicates per test sample.

**Supplementary Table S8: Summary of biomarker of exposure (% change from baseline) data from randomized controlled trial conducted with heated tobacco products**

| Publication number | 1 | 1 | 1 | 2 | 3 | 4 | 5 | 6 | 7 | 8 | 9 | 12 | 12 | 12 |
| --- | --- | --- | --- | --- | --- | --- | --- | --- | --- | --- | --- | --- | --- | --- |
| Affiliation | JT | JT | JT | PMI | PMI | PMI | PMI | PMI | PMI | PMI | JT | JT | JT | JT |
| Study location | JP | JP | JP | PL | PL | JP | PL | PL | JP | US | JP | JP | JP | JP |
| Product Name | DT3.0a | THS | mDT3.0a | CHTP 1.2 | CHTP | THS 2.2 | THS 2.2 | THS 2.1 | mTHS | mTHS | NTV | IT2.0a | DT2.2a | THP |
| (Reference product) | (Cig) | (Cig) | (mCig) | (Cig) | (Cig) | (Cig) | (Cig) | (Cig) | (mCig) | (mCig) | (Cig) | (Cig) | (Cig) | (Cig) |
| End of the study (days) | 5 d | 5 d | 5 d | 5 d | 5 d | 5 d | 5 d | 5 d | 5 d | 5 d | 5 d | 5 d | 5 d | 5 d |
| 3-HPMA | -68.53 | -67.16 | -75.75 | -35.02 | -70.60 | -47.33 | -49.68 | -66.90 | -54.35 | -60.63 | -53.00 | -74.88 | -82.47 | -76.09 |
| CEMA | -87.74 | -86.73 | -88.79 | -85.67 | nd | -79.42 | -86.10 | -85.63 | -83.49 | -84.12 | -87.21 | -89.27 | -86.91 | -87.77 |
| 4-ABP | -90.00 | -89.62 | -88.60 | -86.26 | -74.80 | -74.08 | -82.12 | -57.12 | -78.88 | -83.64 | -86.56 | -85.94 | -86.40 | -86.69 |
| 1-AN | -97.43 | -96.65 | -96.12 | -95.74 | nd | -93.12 | -94.16 | nd | -94.89 | -95.91 | -93,94 | -92.77 | -94.27 | -93.31 |
| 2-AN | -89.35 | -89.95 | -91.24 | -88.98 | -79.70 | -75.84 | -85.39 | -87.13 | -87.28 | -87.97 | -90,71 | -90.80 | -92.38 | -91.15 |
| S-PMA | -94.15 | -94.90 | -94.80 | -82.83 | -82.20 | -77.24 | -92.03 | -90.60 | -88.82 | -91.15 | -89.51 | -94.57 | -95.81 | -94.44 |
| 3-OH-BaP | -49.71 | -64.46 | -69.24 | -73.08 | nd | -64.76 | -71.43 | nd | -75.25 | -75.27 | -61.65 | -76.26 | -83.75 | -75.46 |
| MHBMA | -93.50 | -93.28 | -93.44 | -83.04 | -87.60 | -66.41 | -84.98 | -86.72 | -87.50 | -92.02 | -89.68 | -92.52 | -93.44 | -89.77 |
| eCO | -80.63 | -78.21 | -80.94 | nd | nd | nd | nd | nd | nd | nd | -85.08 | -85.55 | -87.24 | -82.40 |
| 3-HMPMA | -77.93 | -81.28 | -83.73 | -76.71 | nd | -60.61 | -80.58 | nd | -58.51 | -67.98 | nd | -46.96 | -72.37 | -54.28 |
| HEMA | -62.50 | -71.05 | -68.00 | -50.82 | nd | -50.99 | -60.71 | nd | -64.48 | -69.06 | -74.10 | -65.83 | -71.68 | -73.29 |
| Total NNAL | -42.35 | -57.34 | -44.82 | -60.19 | -44.70 | -48.04 | -53.98 | -64.35 | -55.74 | -61.97 | -62.67 | -66.20 | -45.86 | -21.47 |
| Total NNN | -50.00 | -74.58 | -40.85 | -77.39 | nd | -59.81 | -69.75 | -85.27 | -73.03 | -86.89 | -89.22 | -93.69 | -71.60 | -26.53 |
| o-Toluidine | -76.23 | -77.71 | -83.03 | -58.48 | -50.40 | -44.23 | -50.96 | -30.88 | -59.71 | -56.99 | -71.87 | -70.70 | -84.50 | -55.60 |
| Total 1-OHP | -22.40 | -36.29 | -50.02 | -52.61 | -46.80 | -58.57 | -60.17 | -63.02 | -69.89 | -55.73 | -10.54 | -44.72 | -49.70 | -30.28 |

| Publication number | 12 | 14 | 10 | 11 | 11 | 11 | 13 | 15 | 15 | 24 | 24 | 17 | 16 | 18 |
| --- | --- | --- | --- | --- | --- | --- | --- | --- | --- | --- | --- | --- | --- | --- |
| Affiliation | JT | PMI | PMI | BAT | BAT | BAT | PM USA | PMI | PMI | PMI | PMI | PMI | PMI | JT |
| Study location | JP | PL | JP | JP | JP | JP | – | JP | JP | UK | UK | KR | PL | JP |
| Product Name | THS | CHTP1.0 | EHCSS K6 | glo/THP | mglo/  THP | iQOS/  THS | EHCSS | EHCSS K3 | EHCSS K6 | EHCSS K3 | EHCSS K6 | EHCSS K3 | EHCSS K6 | HC |
| (Reference product) | (Cig) | (Cig) | (mCig) | (Cig) | (mCig) | (Cig) | (Cig) | (Cig) | (Cig) | (Cig) | (Cig) | (Cig) | (Cig) | (Cig) |
| End of the study (days) | 5 d | 5 d | 6 d | 6-7d | 6-7d | 6-7d | 8 d | 8 d | 8 d | 8 d | 8 d | 8 d | 28 d | 28 d |
| 3-HPMA | -71.81 | -53.00 | -27.90 | -52.95 | -48.74 | -37.42 | -40.10 | -23.10 | -24.20 | -41.20 | -35.50 | - 1.30 | -21.22 | -37.14 |
| CEMA | -87.66 | -83.30 | nd | -89.24 | -87.81 | -87.17 | nd | nd | nd | nd | nd | nd | nd | nd |
| 4-ABP | -85.44 | -76.50 | -40.80 | -80.57 | -81.90 | -78.26 | -59.80 | -53.40 | -48.60 | nd | nd | nd | -51.03 | -68.55 |
| 1-AN | -91.71 | -95.80 | nd | nd | nd | nd | nd | nd | nd | nd | nd | nd | nd | nd |
| 2-AN | -89.35 | -87.70 | nd | -90.63 | -90.19 | -89.95 | -66.10 | -9.80 | -14.60 | nd | nd | nd | -51.60 | nd |
| S-PMA | -93.28 | -84.20 | -83.40 | -89.13 | -92.48 | -89.78 | -76.70 | -71.00 | -75.60 | -83.10 | -79.40 | -40.10 | -64.35 | -40.09 |
| 3-OH-BaP | -78.16 | -72.40 | nd | nd | nd | nd | nd | nd | nd | nd | nd | nd | nd | nd |
| MHBMA | -89.66 | -72.60 | -18.90 | -91.32 | -89.48 | -84.30 | -63.80 | -49.50 | -55.30 | -54.40 | -53.80 | -32.40 | -52.99 | -51.30 |
| eCO | -83.88 | nd | nd | -87.25 | -89.62 | -85.33 | nd | nd | nd | nd | nd | nd | nd | nd |
| 3-HMPMA | -54.21 | -72.9 | -58.60 | -78.82 | -80.93 | -76.13 | -52.80 | -38.30 | -41.20 | -54.80 | -52.80 | nd | nd | -56.48 |
| HEMA | -66.99 | -58.3 | nd | -56.46 | -60.72 | -59.63 | nd | nd | nd | nd | nd | nd | nd | nd |
| Total NNAL | -56.04 | -52.70 | -55.20 | -35.07 | -36.99 | -53.91 | -65.50 | -52.60 | -51.50 | -60.10 | -55.20 | -50.50 | -46.20 | -53.35 |
| Total NNN | -71.16 | -59.20 | nd | -49.35 | -51.90 | -88.39 | nd | nd | nd | nd | nd | nd | nd | nd |
| o-Toluidine | -68.76 | -65.50 | -53.30 | -48.78 | -63.15 | -49.23 | -15.80 | -73.00 | -68.40 | -66.20 | -61.70 | -61.80 | -39.01 | nd |
| Total 1-OHP | -42.16 | -55.30 | -67.70 | -64.23 | -73.49 | -78.78 | -62.70 | -66.70 | -66.70 | -64.00 | -63.20 | -38.20 | -26.70 | -41.83 |

| Publication number | 20 | 8 | 7 | 2 | 19 | 23 | 21 | 19 | 23 | 22 | 23 |
| --- | --- | --- | --- | --- | --- | --- | --- | --- | --- | --- | --- |
| Affiliation | PM USA | PMI | PMI | PMI | PMI | BAT | RAI, RJR | PMI | BAT | PM USA | BAT |
| Study location | – | US | JP | PL | US | UK | US | US | UK | – | UK |
| Product Name | EHCSS | mTHS | mTHS | CHTP 1.2 | THS 2.2 | glo/THP | Eclipse | THS 2.2 | glo/THP | EHCSS | glo/THP |
| (Reference product) | (Cig) | (mCig) | (mCig) | (Cig) | (Cig) | (Cig) | (Cig) | (Cig) | (Cig) | (Cig) | (Cig) |
| End of the study (days) | 84 d | 90 d | 90 d | 90 d | 90 d | 90 d | 168 d | 180 d | 180 d | 360 d | 360 d |
| 3-HPMA | -25.00 | -57.54 | -42.11 | -59.86 | -29.98 | -62.00 | 20.00 | -24.16 | -65.00 | -35.00 | -60.00 |
| CEMA | nd | -84.68 | -89.49 | -89.97 | -49.13 | -82.00 | nd | -49.06 | -84.00 | nd | -83.00 |
| 4-ABP | nd | -67.10 | -77.81 | -70.61 | nd | -69.00 | -64.00 | nd | -74.00 | -43.00 | -71.00 |
| 1-AN | nd | -84.82 | -94.22 | -91.53 | nd | nd | nd | nd | nd | nd | nd |
| 2-AN | nd | -82.32 | -84.89 | -86.34 | nd | -77.00 | -66.00 | nd | -79.00 | nd | -79.00 |
| S-PMA | -48.60 | -78.77 | -86.25 | -78.90 | nd | -80.00 | -51.00 | nd | -83.00 | nd | -82.00 |
| 3-OH-BaP | nd | -61.02 | -64.14 | -59.02 | -29.81 | nd | nd | -27.95 | nd | nd | nd |
| MHBMA | nd | -81.74 | -78.31 | -74.84 | -44.14 | -76.00 | -56.00 | -40.38 | -83.00 | nd | -77.00 |
| eCO | nd | nd | nd | nd | -31.51 | -78.00 | nd | -23.74 | -77.00 | nd | -77.00 |
| 3-HMPMA | nd | -66.39 | -48.57 | -70.71 | -34.65 | -70.00 | -34.00 | -28.05 | -71.00 | nd | -72.00 |
| HEMA | nd | -61.50 | -45.64 | -44.66 | nd | -43.00 | nd | nd | -81.00 | nd | -76.00 |
| Total NNAL | -62.60 | -69.40 | -72.87 | -75.03 | -41.22 | -55.00 | -39.00 | -46.28 | -43.00 | -73.00 | -66.00 |
| Total NNN | nd | -87.94 | -68.53 | -82.81 | -37.80 | -48.00 | nd | -41.47 | -37.00 | nd | -20.00 |
| o-Toluidine | nd | -51.98 | -46.68 | -60.64 | nd | -62.00 | -36.00 | nd | -65.00 | nd | -60.00 |
| Total 1-OHP | 17.50 | -26.51 | -44.49 | -31.21 | -22.34 | -51.00 | 25.00 | -21.31 | -44.00 | -53.00 | -42.00 |

Table S8 presents data on percent change in BoE levels following HTP use among adult smokers compared to baseline, as reported by or calculated from data extracted from the references (baseline and end of study). Acronyms for BoEs with corresponding parent compound in aerosol: 3-HPMA, 3-hydroxypropyl-mercapturic acid (acrolein); CEMA, 2-cyanoethylmercapturic acid (acrylonitrile); 4-ABP, 4-Aminobiphenyl (4-Aminobiphenyl); 1-AN, 1-Aminonaphthalene (1-Aminonaphthalene); 2-AN, 2-Aminonaphthalene (2-Aminonaphthalene); S-PMA, S-phenylmercapturic acid (benzene); 3-OH-BaP, 3-hydroxy-benzo[a]pyrene (benzo[a]pyrene); MHBMA, monohydroxybutenyl-mercapturic acid (1,3-butadiene); eCO, exhaled carbon monoxide (carbon monoxide); 3-HMPMA, 3-hydroxy-3-methylpropylmercapturic acid (crotonaldehyde); HEMA, 2-hydroxyethyl-mercapturic acid (ethylene oxide); Total NNAL, Total 4-(methylnitrosamino)-1-(3-pyridyl)-1-butanol, (4-(methylnitrosamino)-1-(3-pyridyl)-1-butanone [NNK]); Total NNN, Total N-nitrosonornicotine (N-nitrosonornicotine [NNN]); o-Toluidine (o-Toluidine); Total 1-OHP, Total 1-hydroxypyrene (pyrene).

References mentioned in the table are listed below;

Nishihara, D., *et al.* A Randomized Control Study in Healthy Adult Smokers to Assess Reduced Exposure to Selected Cigarette Smoke Constituents in Switching to the Novel Heated Tobacco Product DT3.0a*.* *Clin Pharmacol Drug Dev* **2024**, *13*(1), p. 45-57.

Bosilkovska, M., *et al.* Exposure to harmful and potentially harmful constituents decreased in smokers switching to Carbon-Heated Tobacco Product*.* *Toxicol Lett* **2020**, *330*, p. 30-40.

Ludicke, F., *et al.* Evaluation of Biomarkers of Exposure in Smokers Switching to a Carbon-Heated Tobacco Product: A Controlled, Randomized, Open-Label 5-Day Exposure Study*.* *Nicotine Tob Res* **2016**, *18*(7), p. 1606-13.

Haziza, C., *et al.* Assessment of the reduction in levels of exposure to harmful and potentially harmful constituents in Japanese subjects using a novel tobacco heating system compared with conventional cigarettes and smoking abstinence: A randomized controlled study in confinement*.* *Regul Toxicol Pharmacol* **2016**, *81*, p. 489-499.

Haziza, C., *et al.* Biomarker of exposure level data set in smokers switching from conventional cigarettes to Tobacco Heating System 2.2, continuing smoking or abstaining from smoking for 5 days*.* *Data Brief* **2017**, *10*, p. 283-293.

Ludicke, F., *et al.* Reduced Exposure to Harmful and Potentially Harmful Smoke Constituents With the Tobacco Heating System 2.1*.* *Nicotine Tob Res* **2017**, *19*(2), p. 168-175.

Ludicke, F., *et al.* Effects of Switching to the Tobacco Heating System 2.2 Menthol, Smoking Abstinence, or Continued Cigarette Smoking on Biomarkers of Exposure: A Randomized, Controlled, Open-Label, Multicenter Study in Sequential Confinement and Ambulatory Settings (Part 1)*.* *Nicotine Tob Res* **2018**, *20*(2), p. 161-172.

Haziza, C., *et al.* Reduction in Exposure to Selected Harmful and Potentially Harmful Constituents Approaching Those Observed Upon Smoking Abstinence in Smokers Switching to the Menthol Tobacco Heating System 2.2 for 3 Months (Part 1)*.* *Nicotine Tob Res* **2020**, *22*(4), p. 539-548.

Yuki, D., *et al.* Assessment of the exposure to harmful and potentially harmful constituents in healthy Japanese smokers using a novel tobacco vapor product compared with conventional cigarettes and smoking abstinence*.* *Regul Toxicol Pharmacol* **2018**, *96*, p. 127-134.

Tricker, A.R., *et al.* Reduced exposure evaluation of an Electrically Heated Cigarette Smoking System. Part 6: 6-Day randomized clinical trial of a menthol cigarette in Japan*.* *Regul Toxicol Pharmacol* **2012**, *64*(2 Suppl), p. S64-73.

Gale, N., *et al.* Changes in Biomarkers of Exposure on Switching From a Conventional Cigarette to Tobacco Heating Products: A Randomized, Controlled Study in Healthy Japanese Subjects*.* *Nicotine Tob Res* **2019**, *21*(9), p. 1220-1227.

Yuki, D., *et al.* Assessment of the exposure to selected smoke constituents in adult smokers using in-market heated tobacco products: a randomized, controlled study. *Sci Rep* **2022**, 12(1), p. 18167.

Frost-Pineda, K., *et al*. Short-term clinical exposure evaluation of a third-generation electrically heated cigarette smoking system (EHCSS) in adult smokers*.* *Regul Toxicol Pharmacol* **2008**, *52*(2), p. 104-10.

Tran, C.T., *et al.* Reduced levels of biomarkers of exposure in smokers switching to the Carbon-Heated Tobacco Product 1.0: a controlled, randomized, open-label 5-day exposure trial. *Sci Rep* **2020**, 10(1), p. 19227.

Tricker, A.R., *et al.* Reduced exposure evaluation of an Electrically Heated Cigarette Smoking System. Part 5: 8-Day randomized clinical trial in Japan*.* *Regul Toxicol Pharmacol* **2012**, *64*(2 Suppl), p. S54-63.

Martin Leroy, C., *et al.* Reduced exposure evaluation of an Electrically Heated Cigarette Smoking System. Part 7: A one-month, randomized, ambulatory, controlled clinical study in Poland*.* *Regul Toxicol Pharmacol* **2012**, *64*(2 Suppl), p. S74-84.

Tricker, A.R., *et al*. Reduced exposure evaluation of an Electrically Heated Cigarette Smoking System. Part 4: Eight-day randomized clinical trial in Korea*.* *Regul Toxicol Pharmacol* **2012**, *64*(2 Suppl), p. S45-53.

Sakaguchi, C., *et al.* Exposure evaluation of adult male Japanese smokers switched to a heated cigarette in a controlled clinical setting*.* *Regul Toxicol Pharmacol* **2014**, *69*(3), p. 338-47.

Ludicke, F., *et al.* Effects of Switching to a Heat-Not-Burn Tobacco Product on Biologically Relevant Biomarkers to Assess a Candidate Modified Risk Tobacco Product: A Randomized Trial*.* *Cancer Epidemiol Biomarkers Prev* **2019**, *28*(11), p. 1934-1943.

Frost-Pineda, K., *et al.* 12-week clinical exposure evaluation of a third-generation electrically heated cigarette smoking system (EHCSS) in adult smokers*.* *Regul Toxicol Pharmacol* **2008**, *52*(2), p. 111-7.

Ogden, M.W., *et al.* Switching from usual brand cigarettes to a tobacco-heating cigarette or snus: Part 2. Biomarkers of exposure*.* *Biomarkers* **2015**, *20*(6-7), p. 391-403.

Roethig, H.J., *et al.* A 12-month, randomized, controlled study to evaluate exposure and cardiovascular risk factors in adult smokers switching from conventional cigarettes to a second-generation electrically heated cigarette smoking system*.* *J Clin Pharmacol* **2008**, *48*(5), p. 580-91.

Gale, N., *et al.* Changes in biomarkers of exposure and biomarkers of potential harm after 360 days in smokers who either continue to smoke, switch to a tobacco heating product or quit smoking*.* *Intern Emerg Med* **2022**, *17*(7), p. 2017-2030.

Tricker, A.R., *et al.* Reduced exposure evaluation of an Electrically Heated Cigarette Smoking System. Part 3: Eight-day randomized clinical trial in the UK*.* *Regul Toxicol Pharmacol* **2012**, *64*(2 Suppl), p. S35-44.

**Supplementary Table S9: Summary of smoke or aerosol levels for a subset of analytes**

| **Analyte**  **(Smoke/**  **Aerosol)** | **Reported Data [This Study]**  **Mean** | **This Study**  **Data 1R6F**  **Mean** | **Reported Data on iQOS™ Induction Heating Technology**  **[20]**  **Regular / Menthol** | **Reported Data 1R6F**  **[20]** | **Reported Data on iQOS™ Blade Heating Technology**  **[18]**  **Regular / Menthol** | **Reported Data 3R4F**  **[18]** |
| --- | --- | --- | --- | --- | --- | --- |
| **Acrylamide**  µg/stick | 1.60 | **4.63** | 1.02/ 1.06 | **4,88** | 1.64/ 1.80 | **4.33** |
| **Ammonia**  µg/stick | 7.67 | **31.28** | 10.7/7.57 | **22** | 13.14/ 13.38 | **31.7** |
| **Arsenic**  ng/stick | 1.81 | **7.94** | 0.816/ <0.75 | **7.96** | <1.2/<1.2 | **8.23** |
| **Mercury**  ng/stick | 1.58 | **4.51** | 1.54/ 1.81 | **5.66** | 2.11/ 1.88 | **4.36** |
| **Selenium**  ng/stick | 0.63 | **1.92** | < 0.4 /< 0.4 | **2.08** | <0.83/<0.83 | **< 4.42** |
